# The genus *Clavariadelphus* (Clavariadelphaceae, Gomphales) in China

**DOI:** 10.3897/mycokeys.70.54149

**Published:** 2020-07-29

**Authors:** Hong-Yan Huang, Jie Zhao, Ping Zhang, Zai-Wei Ge, Xian Li, Li-Ping Tang

**Affiliations:** 1 School of Pharmaceutical Sciences and Yunnan Key Laboratory of Pharmacology for Natural Products, Kunming Medical University, Kunming, 650500, China Kunming Medical University Kunming China; 2 College of Life Science, Hunan Normal University, Changsha, 410081, China Hunan Normal University Changsha China; 3 Key Laboratory for Plant Diversity and Biogeography of East Asia, Kunming Institute of Botany, Chinese Academy of Sciences, Kunming, 650201, China Kunming Institute of Botany, Chinese Academy of Sciences Kunming China

**Keywords:** Clavarioid fungi, taxonomy, molecular systematics, new taxa, species diversity

## Abstract

*Clavariadelphus* species (Clavariadelphaceae, Gomphales) in China were examined using morphology, molecular phylogenetic analyses of ITS data and chemical reactions. Eleven taxa were identified in China, including four species known previously to occur in China (*C. griseoclavus*, *C. ligula*, *C. sachalinensis* and *C. yunnanensis*), two new record species from China (*C. elongatus* and *C. himalayensis*), four novel species (*C. alpinus*, *C. amplus*, *C. gansuensis* and *C. khinganensis*) and one species that could not be described due to the paucity of material. Finally, we also provided a taxonomic key for the identification of *Clavariadelphus* species in China.

## Introduction

*Clavariadelphus* Donk (Clavariadelphaceae, Gomphales, Basidiomycota), typified by *C. pistillaris* (L.) Donk, is a group of fungi characterised by erect, simple, club-shaped basidiomes with rhizomorphs at the stipe base, hymenium with (2–) 4-spored basidia, clavate leptocystidia, ellipsoid to amygdaliform, thin-walled, inamyloid basidiospores and clamp connections at the septa of the hyphae (Methven 1990). The genus is widely distributed in temperate regions of the Northern Hemisphere and 24 species were described before this study.

*Clavariadelphus* has been studied in Europe and North America and important taxonomic works are available (Corner 1950, 1970; Welden 1966; Smith and Corner 1967; Petersen 1967, 1972; Smith 1971; Petersen et al. 1974; Methven 1989; Methven and Guzmán 1989). The genus has not received as much attention in Asia, except for a couple of novel species described from Pakistan (Hanif et al. 2014; Sher et al. 2018). In China, two novel taxa have been described (Methven 1989; Lu and Li 2020). To date, only seven *Clavariadelphus* species have been reported in China, namely *C. griseoclavus* L. Fan & L. Xia, *C. ligula* (Schaeff.) Donk, *C. pallido-incarnatus* Methven, *C. pistillaris*, *C. sachalinensis* (S. Imai) Corner, *C. truncatus* Donk and *C. yunnanensis* Methven (Methven 1989, 1990; Mao et al. 1993; Yuan and Sun 1995; Zang 1996; Bau et al. 2003; Mao 2009; Tang and Yang 2014; Tang 2015; Lu and Li 2020). The studies, in which these species were identified, are comparatively brief and solely based on morphological criteria except *C. griseoclavus*.

Although *Clavariadelphus* can be readily distinguished from other members of the Gomphales, the delimitation of infrageneric taxa is difficult in many cases due to subtle variations in morphological characteristics and growth habits (Methven 1990). Recently, molecular techniques have been widely applied and have provided useful information for species delimitation in systematic fungal studies (Hibbett 2007; Yang 2011). Chemical reactions are also helpful in delimiting species of many macrofungal groups besides *Clavariadelphus*, including *Agaricus*, *Boletopsis*, *Chroogomphus*, *Cortinarius*, *Hygrophorus*, *Leucoagaricus* and *Leratiomyces* (Corner 1950; Hanif et al. 2014; Siegel and Schwarz 2016). Scanning electron microscopy (SEM) has been applied to the identification of other macrofungal groups (Zeng et al. 2013; Tang et al. 2014; Huang et al. 2018). However, SEM of structures of *Clavariadelphus* has not yet been reported. We mainly examined Chinese *Clavariadelphus* collections through analysis of morphological characteristics using light microscopy and SEM, as well as molecular phylogenetic data, ecological data and chemical reactions, to better understand species diversity of *Clavariadelphus* in China.

## Materials and methods

### Morphological studies

Aside from one collection from the Czech Republic, most specimens of *Clavariadelphus* in this study were collected from coniferous forests or mixed coniferous and broad-leaved forests in North (N) China, Northwest (NW) China and Southwest (SW) China during the rainy seasons (July–September). Collections and field records are deposited in the Herbarium of Cryptogams, Kunming Institute of Botany, Chinese Academy of Sciences (HKAS), Mycological Herbarium, Institute of Mycology, Chinese Academy of Sciences (HMAS), Mycological Herbarium of Hunan Normal University (MHHNU) and Mycological Herbarium of Pharmacy College, Kunming Medical University (MHKMU) (Appendix 1). Specimens and their habitats were photographed *in situ*. Relevant metadata, such as altitude, latitude, longitude and nearby tree associates were recorded in the field. Detailed notes on macro-morphological descriptions were taken from fresh material and colour codes were from Kornerup and Wanscher (1981).

### Light microscopy

Micro-morphological characteristics were observed under a light microscope (Leica DM 2500). Preparations were made from dried specimens. Tissue fragments of dried materials were sectioned, mounted in 10% KOH and observed. The abbreviation [n/m/p] means n basidiospores measured from m basidiomes of p collections. Dimensions for basidiospores are given as (a) b–c (d). The range of b–c contains a minimum of 90% of the measured values. Extreme values, i.e. a and d are given in parentheses. *Q* is used to denote the length/width ratio of basidiospores in the side view, whereas *Q_m_* refers to the average *Q* value of all basidiospores ± standard deviation.

### Scanning electron microscopy

The material was sampled and directly used from herbarium collections. The hymenium and basal mycelium from dried specimens were mounted on to aluminium stubs coated with gold palladium. Basidiospores and hyphae of the basal mycelium were observed and micrographs were taken with a ZEISS Sigma 300 scanning electron microscope at 7.0 kV accelerating voltage.

### Chemical reactions

Seven chemical reagents were used: 10% (w/v) KOH, 10% (w/v) FeCl_3_, 10% (w/v) FeSO_4_, 10% NH_4_OH, 10% (w/v) phenol, Melzer’s reagent and 95% (v/v) ethanol. Small slices of tissue were taken from the hymenium of the basidiomes. The reagents were systematically added to the depression in plates so that each piece of tissue was submerged in several drops of a single reagent. Positive colour reactions were recorded immediately following the application of reagents.

### DNA extraction, PCR and DNA sequencing

Total genomic DNA was isolated from dried materials using a modified CTAB method (Doyle 1987) with a prolongation of the extraction period as necessary. For PCR reactions, the nuclear ribosomal DNA internal transcribed spacer (ITS) region was amplified using primers ITS5 and ITS4 (White et al. 1990). The PCR amplification mix consisted of a total volume of 25 μl containing 2.5 μl of 10 × amplification buffer (with MgCl_2_), 0.5 μl dNTP (200 μM), 0.2 μl Taq DNA polymerase (5 U/μl), 1 μl of each primer (10 μM), 1 μl DNA template and 18.8 μl sterile water. PCR reactions were performed with an initial denaturation at 94 °C for 4 min; 38 cycles of denaturation at 94 °C for 40 s, annealing at 54 °C for 40 s, extension at 72 °C for 60 s; and a final extension at 72 °C for 8 min. PCR products were checked on 1% agarose gel. Successful reactions were sequenced using an ABI 3730 DNA Analyzer (Sangon, Shanghai, China) with both PCR primers. The DNA sequences were used as queries in NCBI BLAST searches to rule out contamination. The forward and reverse sequences were assembled with SeqMan (DNASTAR Lasergene 9) and their quality controlled according to the guidelines of Nilsson et al. (2012). Novel and already available sequences were aligned by using MAFFT version 7 (Katoh and Standley 2013). The alignment was manually adjusted in BioEdit version 7.0.9 (Hall 1999) and trimmed in trimAl version 1.2 (Capella-Gutiérrez et al. 2009).

### Phylogenetic analyses

Two phylogenetic tree inference methods, Randomised Accelerated Maximum Likelihood (RAxML) and Bayesian Analysis (BA), were used to analyse the ITS sequence data. The programme RAxML version 7.0.3 (Stamatakis et al. 2008) was used to infer a maximum likelihood tree with bootstrap support values and the GTRGAMMA was selected as a default model. The programme MrBayes version 3.2.6 (Ronquist et al. 2012) was run using a Markov Chain Monte Carlo (MCMC) tree sampling procedure. The ITS1, 5.8S and ITS2 loci were extracted from the aligned ITS dataset, allowing the selection of substitution models for each partition. Aligned sequences were partitioned into ITS1 (1–270), 5.8S (271–429) and ITS2 (430–703). Nucleotide substitution models based on the Akaike Information Criteria (AIC) data were obtained in PartitionFinder 2 (Lanfear et al. 2016). The selected models were GTR+G for ITS1, K80 for 5.8S and HKY+G for ITS2. After four simultaneous Markov chains running with 7,000,000 generations and sampling every 100 generations, the average deviation of split frequencies was 0.004022 at the end of the run. Burn-in values were determined in Tracer v1.7 (Rambaut et al. 2018). Effective sample sizes were well over 200 for all sampled parameters for each run and the initial 20% of the samples was discarded. Bayesian Posterior Probabilities (PP) were calculated for a majority consensus tree of the retained Bayesian trees.

## Results

### Taxonomic identification based on morphological data

Fifty specimens of *Clavariadelphus* were examined in this study. Six species were previously reported from China, except the late described one, *C. griseoclavus*. However, the re-examination of available vouchers confirmed the occurrence of only three of these species, specifically *C. ligula*, *C. sachalinensis* and *C. yunnanensis*. Our morphological observations revealed that nine taxa, including three species previously identified in China (*C. ligula*, *C. sachalinensis* and *C. yunnanensis*), two species that have not been previously reported from China (*C. elongatus* and *C. himalayensis*) and four novel species (*C. alpinus*, *C. amplus*, *C. gansuensis* and *C. khinganensis*), were identified on the basis of morphological characters. So far, there are ten described taxa in China, including *C. griseoclavus* which is recently published.

### Taxonomic identification based on molecular data

The ITS dataset comprised 27 ingroup taxa including the type species *C. pistillaris* and three outgroup taxa, with 64 sequences in total. The length of the alignment was 703 aligned bases (TreeBASE accession 24163). Three species of *Lentaria* Corner and *Kavinia* Pilát were chosen as outgroups in the dataset, based on a previous study (Giachini et al. 2010).

**Figure 1.**
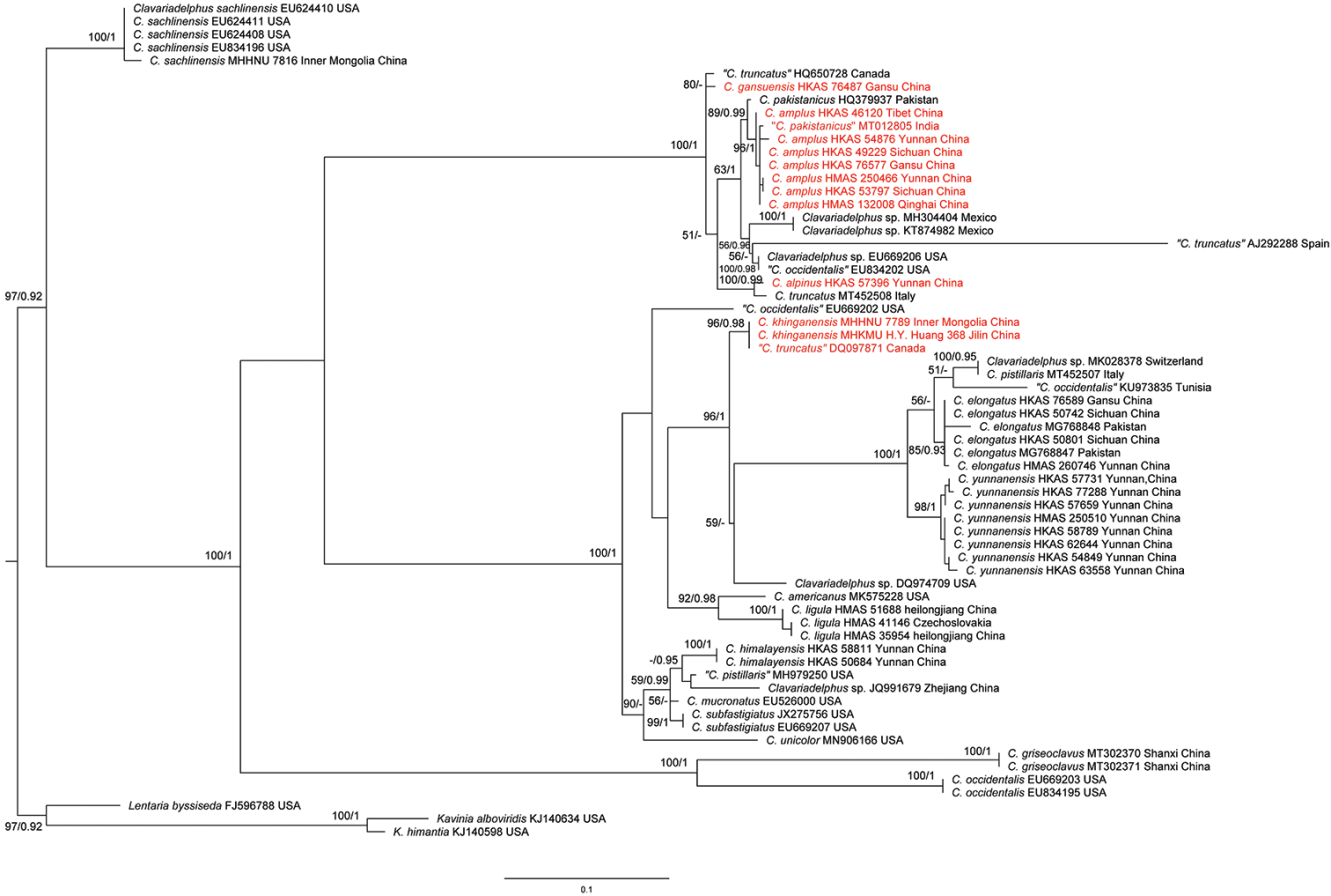
Phylogenetic tree of *Clavariadelphus* based on ITS sequence data. RAxML BP values (≥ 50%) are shown above branches, Bayesian posterior probabilities (≥ 0.90) are shown above branches; new taxa are marked in red.

In the phylogeny, based on ITS sequences, few differences in the topology of major clades were detected between the ML and Bayesian analyses. Twenty-seven phylogenetic species were recovered, amongst which, eleven species were from China, including one with a GenBank accession JQ991679 from Zhejiang Province, China, which might represent a separate species in the tree (Fig. 1). *Clavariadelphus sachalinensis* formed a distinct lineage with high support and was sister to the rest of the genus. Seven Chinese lineages, namely *C. amplus*, *C. elongatus*, *C. griseoclavus*, *C. himalayensis*, *C. ligula*, *C. khinganensis* and *C. yunnanensis*, were strongly supported as monophyletic groups. The other two species from China, namely *C. alpinus* and *C. gansuensis*, were each represented by only one specimen in the phylogenetic tree. The sister of each Chinese taxon is discussed below.

### Taxonomic identification based on chemical reactions

Steglich et al. (1984) proposed that a positive ferric salts reaction of the basidiomes was indicative of the presence of pistillarin in the basidiomes of *Clavariadelphus*. To a large extent, Methven’s study (1990) supported this hypothesis, excluding one exception (*C. cokeri* V.L. Wells & Kempton). Methven (1990) mentioned the negative ferric salts reaction of some species might be the result of pistillarin being present in too low concentrations or the result of samples affected by pesticides during storage. In our study, most species have positive reactions with four reagents (FeCl_3_, KOH, NH_4_OH and phenol), but all species from China showed a negative reaction to FeSO_4_, Melzer’s reagent and ethanol. The results of the chemical testing in this study are summarised in Table 1. As those specimens are preserved, pesticides are used regularly. Thus, we agree with Methven’s argument (1990).

**Table 1.** Chemical reactions of representative species of *Clavariadelphus* from China.

Taxa	KOH	FeCl_3_	NH_4_OH	Phenol	Ethanol	Melzer’s reagent	FeSO_4_
*C. alpinus*	3B8	–	6A8	–	–	–	–
*C. amplus*	12A4	1A8	2A8	2A5	–	–	–
*C. elongatus*	2A5	1A8	6A8	–	–	–	–
*C. gansuensis*	9B7	1A8	2A8	2A8	–	–	–
*C. himalayensis*	5B7	30A8	6A8	–	–	–	–
*C. khinganensis*	2A5	–	–	–	–	–	–
*C. ligula*	3B8	–	6A8	–	–	–	–
*C. sachalinensis*	2A5	30A8	6A8	–	–	–	–
*C. yunnanensis*	5B7	30A8	2A8	2A5	–	–	–

Note: “–” indicates negative reactions.

## Taxonomy

### 1. Clavariadelphus alpinus

Taxon classificationFungiGomphalesClavariadelphaceae

J. Zhao & L.P. Tang sp. nov.

C55B570F-668E-5939-BAAC-388E1485E720

MycoBank No: 830258

**Figure 2.**
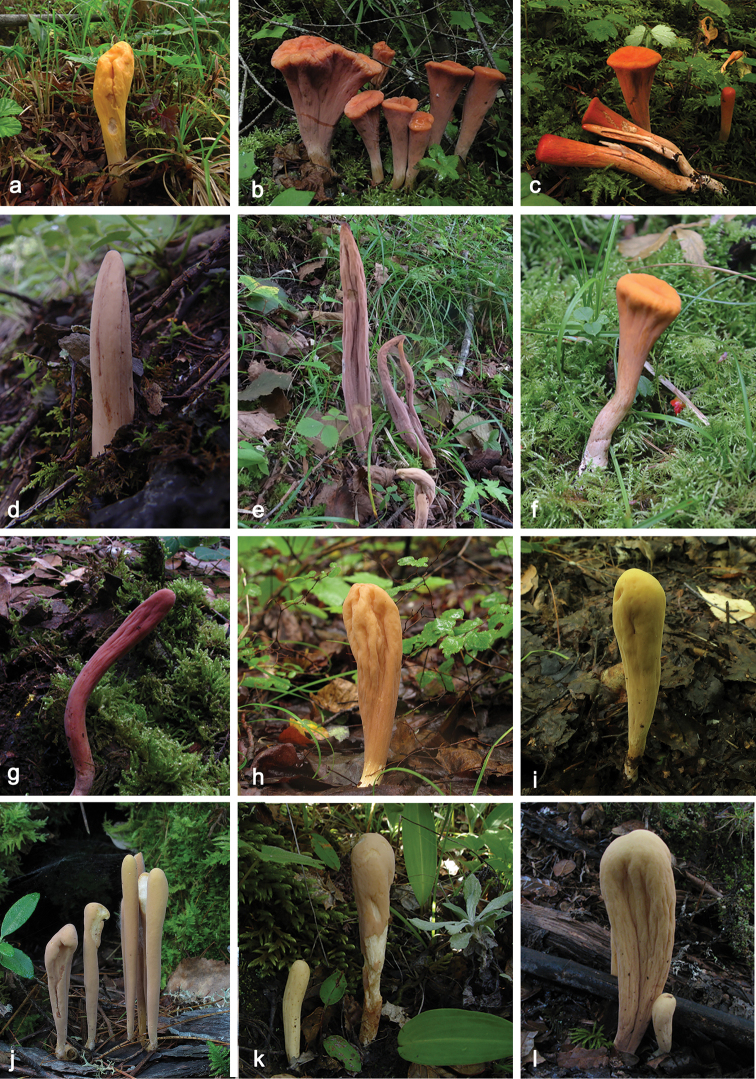
*Clavariadelphus* species in China. **a***C. alpinus* (HKAS 57396, holotype) **b, c***C. amplus* (HKAS 54876, holotype) **d, e***C. elongatus* (**d** from HKAS 50742 **e** from HKAS 76589) **f***C. gansuensis* (HKAS 76487, holotype) **g***C. himalayensis* (HKAS 58811) **h, i***C. khinganensis* (**h** from MHHNU 7789, holotype **i** from MHKMU H.Y. Huang 368) **j***C. sachalinensis* (MHHNU 7816) **k, l***C. yunnanensis* (**k** from HKAS 49398 **l** from HKAS 58789).

**Figure 3.**
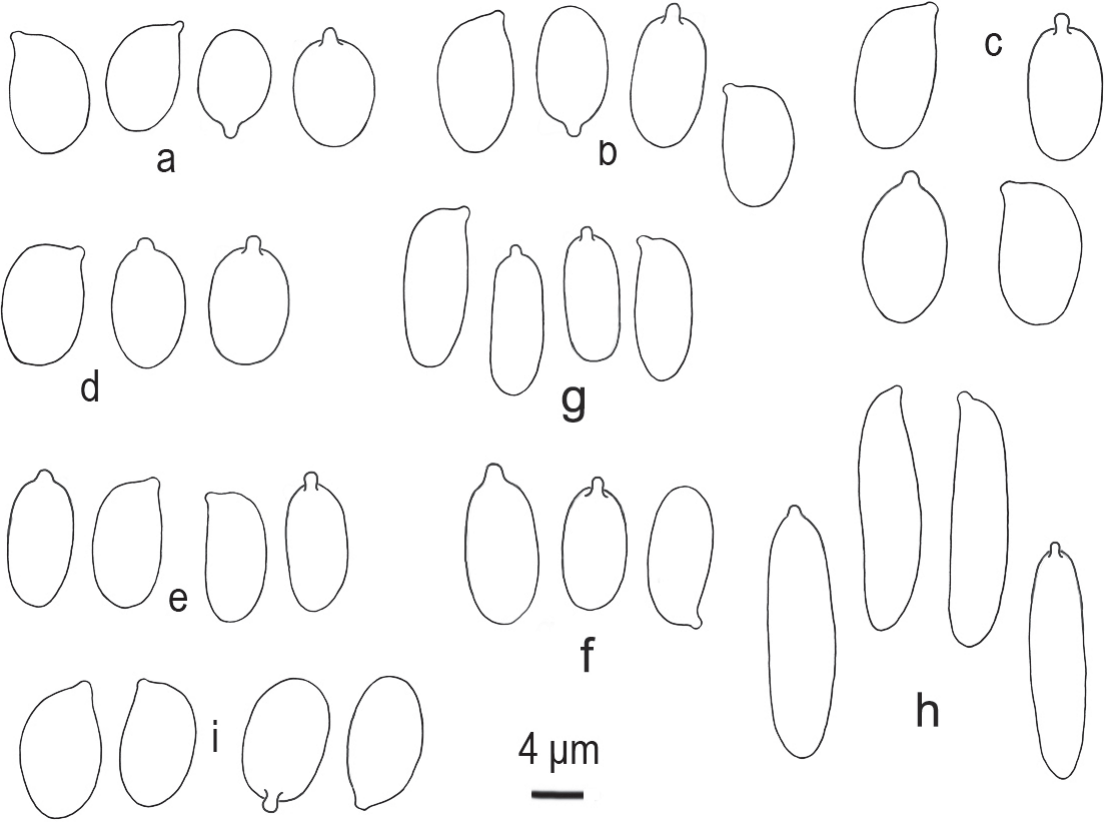
Basidiospores of *Clavariadelphus* under light microscope. **a***C. alpinus* (HKAS 57396, holotype) **b***C. amplus* (HKAS 54876, holotype) **c***C. elongatus* (HKAS 76589) **d***C. gansuensis* (HKAS 76487, holotype) **e***C. himalayensis* (HKAS 58811) **f***C. khinganensis* (MHHNU 7789, holotype) **g***C. ligula* (HKAS 35954) **h***C. sachalinensis* (MHHNU 7816) **i***C. yunnanensis* (HKAS 57659).

**Figure 4.**
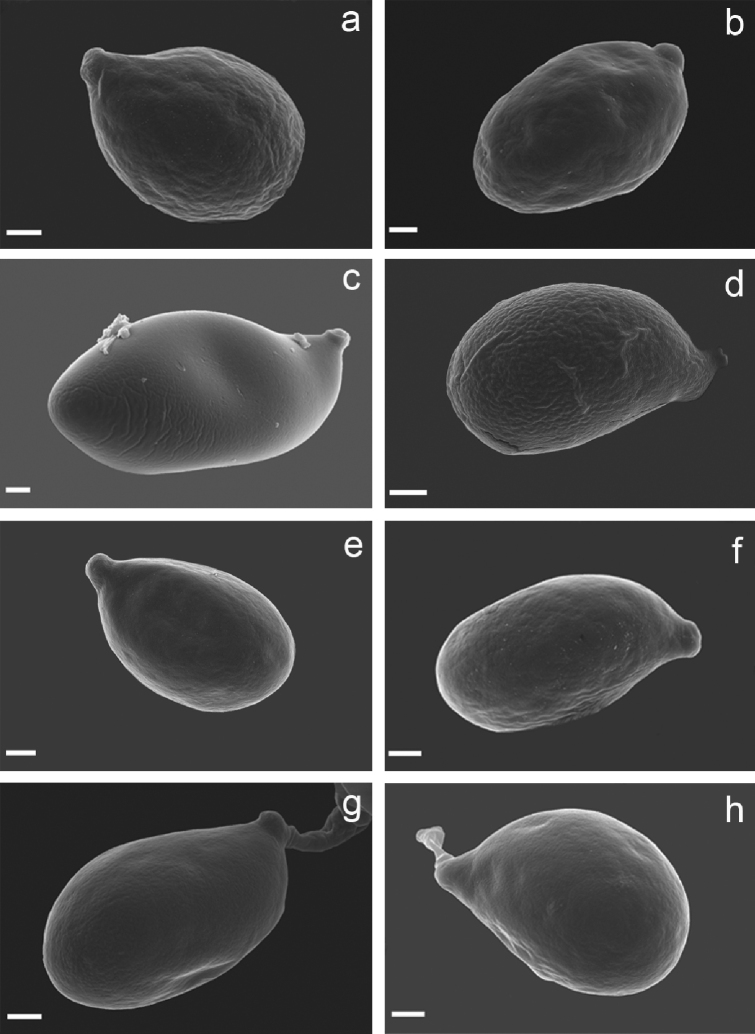
Basidiospores of *Clavariadelphus* under SEM. **a***C. alpinus* (HKAS 57396, holotype) **b***C. amplus* (HKAS 54876, holotype) **c***C. elongatus* (HKAS 76589) **d***C. gansuensis* (HKAS 76487, holotype); **e, f***C. himalayensis* (HKAS 58811) **g***C. khinganensis* (MHHNU 7789, holotype) **h***C. yunnanensis* (HKAS 57659).

**Figure 5.**
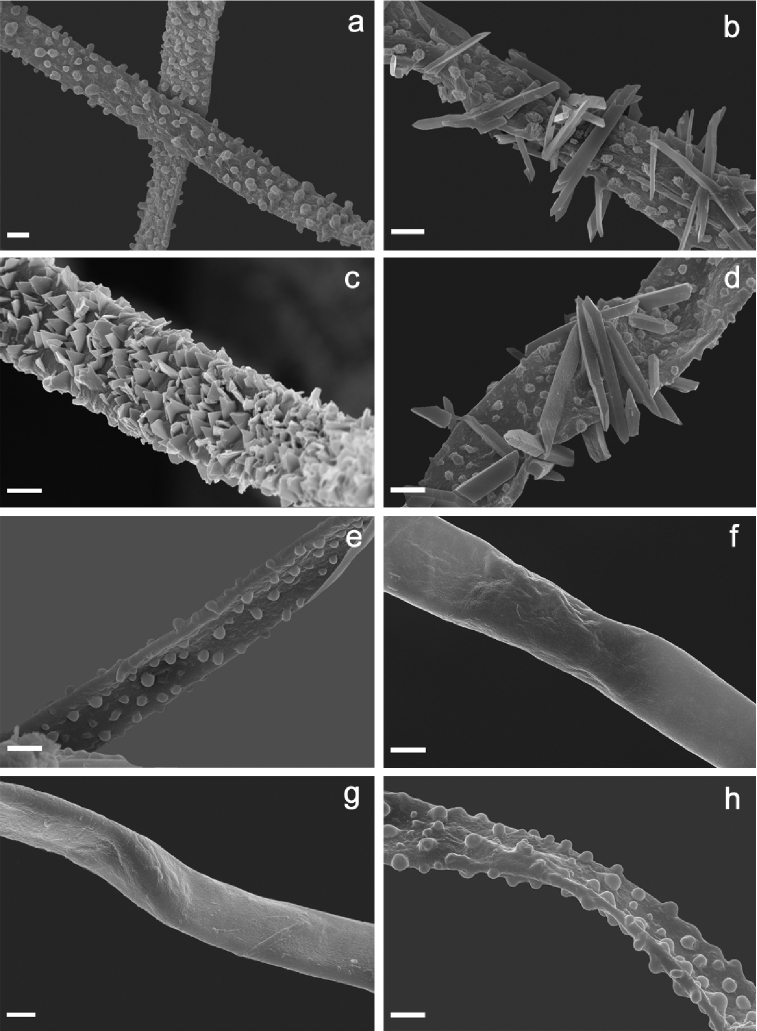
Hyphae of basal mycelium from *Clavariadelphus* under SEM. **a***C. alpinus* (HKAS 57396, holotype) **b***C. amplus* (HKAS 54876, holotype) **c***C. elongatus* (HKAS 76589) **d***C. gansuensis* (HKAS 76487, holotype) **e***C. himalayensis* (HKAS 58811) **f, g***C. sachalinensis* (**f** from HKAS 33844; **g** from MHHNU 7816) **h***C. yunnanensis* (HKAS 57659).

**Figure 6.**
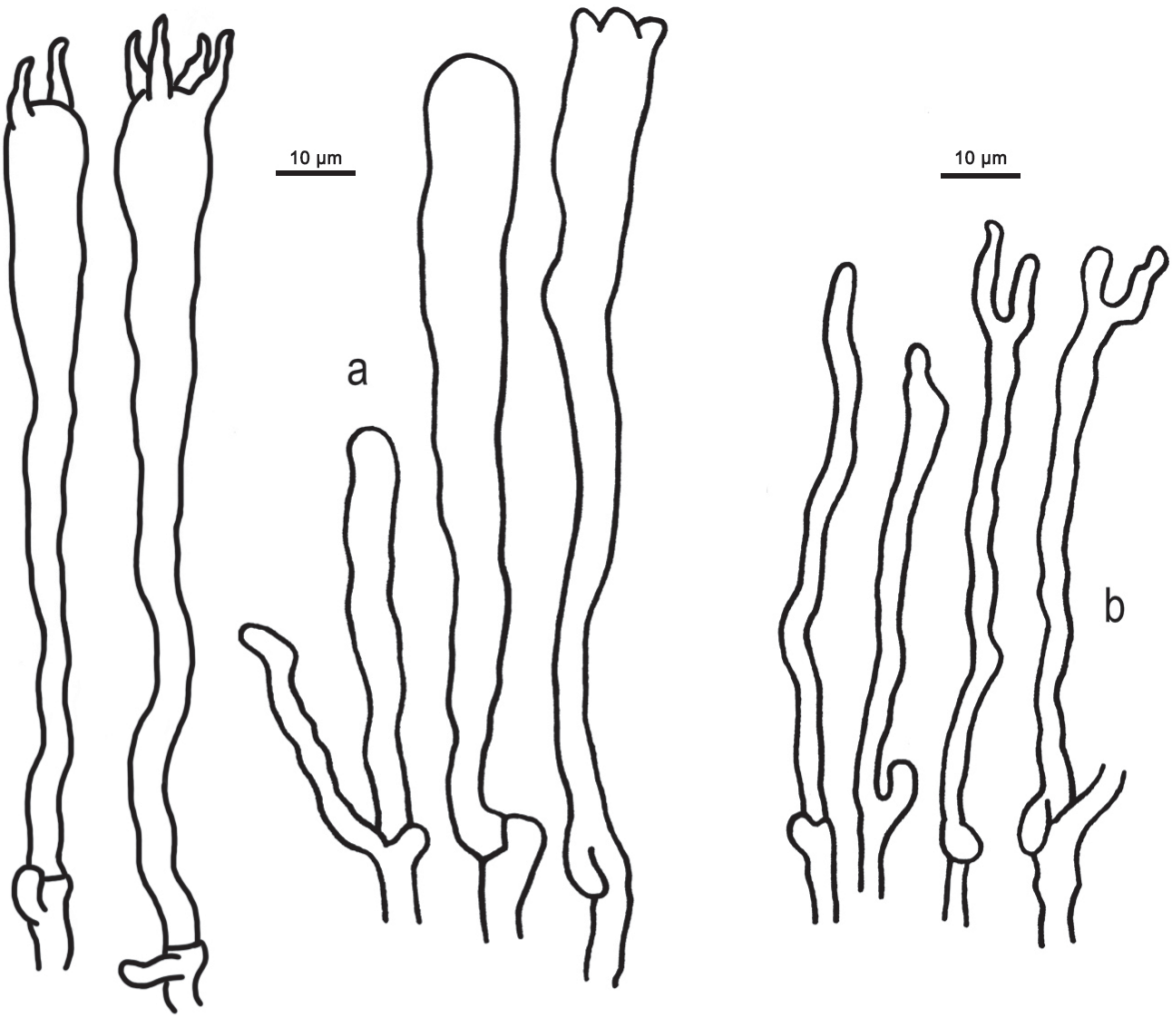
Microscopic features of *Clavariadelphus alpinus* (HKAS 57396, holotype). **a** Basidia **b**Leptocystidia.

Figs 2a , 3a , 4a , 5a , 6a, b

#### Diagnosis.

This species is distinguished from other taxa in *Clavariadelphus* by the light yellow, clavate basidiomes with enlarged apex, broadly ellipsoid basidiospores, hyphae of the basal mycelium with nipple-shaped protuberances and basidiomes turning lemon-chiffon in KOH.

#### Etymology.

Latin “*alpinus*” refers to this species occurring in high-altitude areas.

#### Description.

*Basidiomes* up to 12 cm high, 0.9 cm diam. at the base, enlarged upwards to 2 cm diam., simple, initially cylindrical to subcylindrical, then narrowly clavate to clavate, laterally compressed in age; *hymenium* initially smooth, then longitudinally rugose, light yellow (4A4–5) to yellow or yellowish-orange, apricot-yellow, light orange-yellow (4A6–7) or (5A5–6); *apex* subacute to obtuse, smooth to rugose, concolorous with the hymenium; surface not staining when cut or bruised; *base* terete, smooth, white to cream; *mycelial hyphae* white; *flesh* initially solid, then soft and spongy upwards as the apex enlarges, white not staining on exposure. *Odour* and *taste* not recorded. *Spore deposit* not recorded.

*Hymenium* extending over the apex of basidiomata, composed of basidia and leptocystidia. *Basidia* 65–85 × 8–10 μm, clavate, hyaline, thin-walled, (2–, 3–) 4-spored, sterigmata 8–12 μm in length. *Basidiospores* [20/1/1] (7.4–) 7.8–9.6 (–10.1) × 5.5 (–5.1)–7.4 μm, *Q* = 1.25–1.55 (–1.58), *Q*_m_ = 1.38 ± 0.10, broadly ellipsoid, ovate or amygdaliform, with a small apiculus, inamyloid, thin-walled, hyaline in KOH, smooth. *Leptocystidia* 45–55 × 2.8–4.2 μm, scattered amongst and scarcely projecting beyond the basidia, cylindrical to narrowly clavate, thin-walled, smooth, hyaline, non-pigmented, clamped, inflated apically at maturity and at times, with apical or subapical branches. *Mycelial hyphae* 2–4 μm diam., interwoven or aggregated into rhizomorphic strands, branched, clamped; hyphal walls echinulate with light microscopy, covered with massive nipple-shaped protuberances without crystals with SEM.

#### Chemical reactions

(dried basidiomes). KOH = positive, lemon-chiffon; NH_4_OH = positive, orange; ethanol, FeCl_3_, FeSO_4_, Melzer’s reagent and phenol = negative.

#### Known distribution and ecology.

SW China, Yunnan Province. Solitary on the ground in forests dominated by conifers (e.g. *Abies georgei*) at elevations of approximately 3700 m.

#### Materials examined.

China. Yunnan Province: Shangri-la Prefecture, Bita Lake, 24 August 2009, approximately 3700 m elev., *B. Feng 667* (HKAS 57396, ***Holotype***).

#### Comments.

*Clavariadelphus alpinus* is well characterised by its yellow basidiomes, broadly ellipsoid basidiospores, hyphae of the basal mycelium with nipple-shaped protuberances, the apex of the basidiomes having a positive reaction to NH_4_OH and KOH and distribution at high elevations in SW China in association with conifers.

Morphologically, this taxon is similar to *C. khinganensis*. However, *C. khinganensis* has light brown-tan basidiomes, more elongated basidiospores (*Q* = 1.6–2.2), negative reaction to NH_4_OH and distribution at lower elevations in NE China.

In the ITS phylogeny, this species is a sister species of *C. truncatus* with strong support (Fig. 1). However, *C. truncatus* differs from *C. alpinus* by having dark coloured basidiomes from yellow to cinnamon-brown or brown, broader apices (up to 3.5 cm) and larger basidiospores (10.3–12.6 × 5.5–7.1 μm from neotype; Methven 1990).

### 2. Clavariadelphus amplus

Taxon classificationFungiGomphalesClavariadelphaceae

J. Zhao, L.P. Tang & Z.W. Ge sp. nov.

7E4AB4F0-C743-5038-9598-D8BEEFF384DC

MycoBank No: 830271

**Figure 7.**
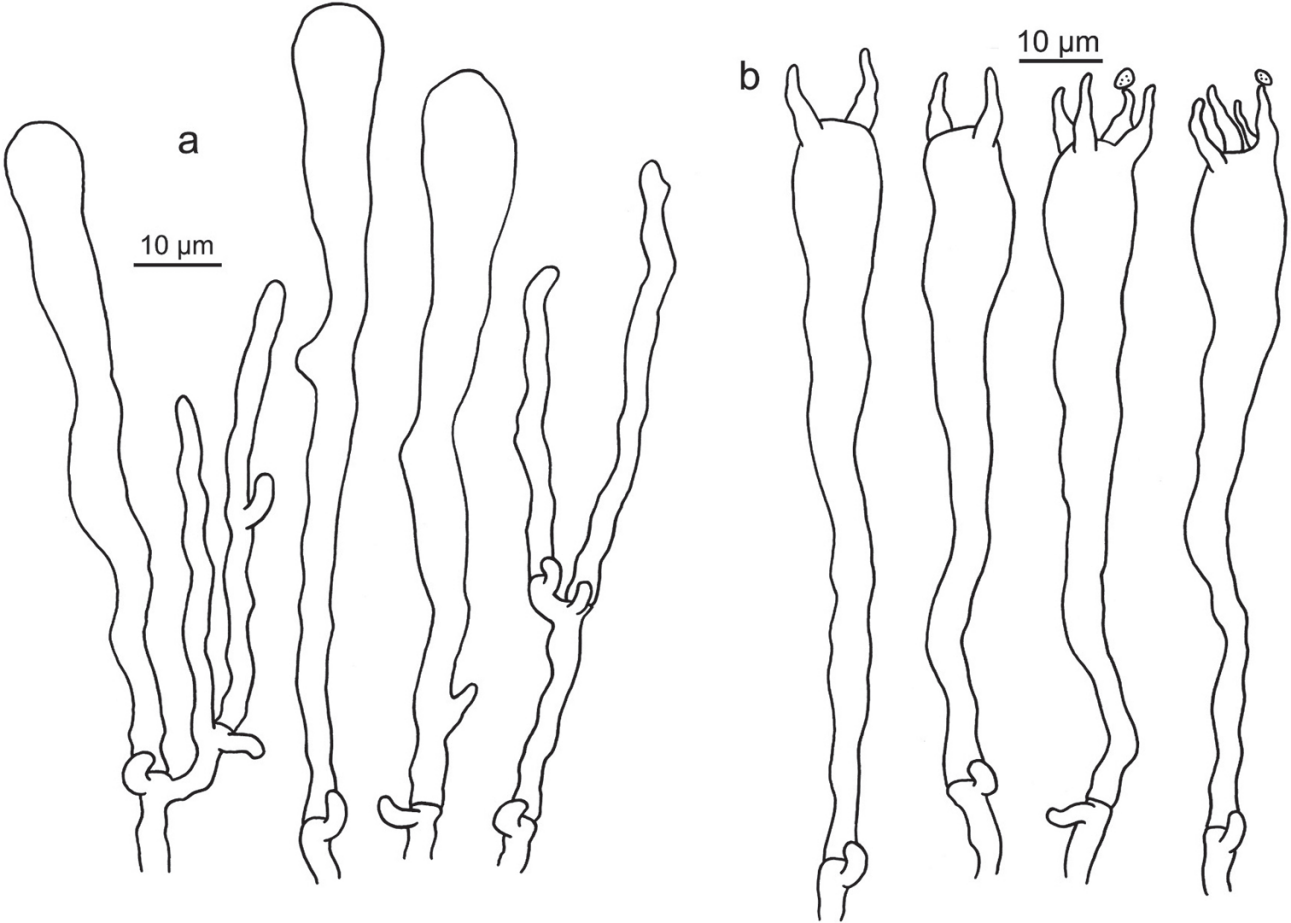
Microscopic features of *Clavariadelphus amplus* (HKAS 54876, holotype). **a**Leptocystidia and immature basidia **b** Basidia.

Figs 2b, c , 3b , 4b , 5b , 7a, b.

#### Diagnosis.

This species is unique in its pink-orange basidiomes with enlarged, truncate and sterile apices, ellipsoid basidiospores, hyphae of the basal mycelium with nipple-shaped protuberances and prism-like crystals and basidiomes turning cherry-red in KOH. It differs from *C. truncatus* by the latter’s darker coloured basidiomes, narrower apices and larger basidiospores.

#### Etymology.

Latin “*amplus*” refers to the enlargement of the apex of the basidiomes.

#### Description.

*Basidiomes* up to 15 cm high, 0.5–1 cm diam. at the base, enlarged upwards to 3–7.5 cm diam. near apex; *hymenium* initially smooth, longitudinally rugulose in age, pruinose, pinkish-orange (7A5–7), paler downwards, greyish-orange (5B4–5); *apex* initially obtuse or broadly rounded, finally truncate, depressed, surface rugose to rugulose, more or less darker than the hymenium, apricot-yellow (5B6–7) to pink-orange, reddish-orange (7A7–8) or red-orange (7B7–8) at maturity; surface slowly staining light brown or light leather-brown (7D6–7) to brown (7E6–7) when cut or bruised, staining more conspicuously downwards; *base* simple, terete, nearly smooth, cylindrical to subcylindrical, pruinose; *mycelial hyphae* interwoven, white; *flesh* solid initially, then soft and spongy upwards as the apex enlarges, white, slowly staining light leather-brown (7D6–7) to brown (7E6–7) on exposure. *Odour* pleasant. *Taste* not distinctive. *Spore deposit* not recorded.

*Hymenium* limited to the sides of basidiomes, composed of basidia and leptocystidia; the apex of basidiomata is composed of sterile elements 18–28 × 5–8 μm, clavate, thin-walled, smooth, clamped. *Basidia* 85–95 × 8–12 μm, clavate, hyaline, thin-walled, (2–) 4-spored, sterigmata 9–11 μm in length. *Basidiospores* [40/2/2] 8.2–11.0 × 5.1–6.4 μm, *Q* = (1.36–) 1.38–2.00 (–2.18), *Q*_m_ = 1.75 ± 0.17, ellipsoid to broadly ellipsoid, ovate or amygdaliform, with a small apiculus, inamyloid, thin-walled, hyaline in KOH, smooth. *Leptocystidia* 45–70 × 2.8–3.8 μm, scattered amongst and scarcely projecting beyond the basidia, cylindrical to narrowly clavate, thin-walled, smooth, hyaline, non-pigmented, clamped, inflated apically at maturity and at times, with apical or subapical branches. *Mycelial hyphae* 2–4 μm diam., parallel, interwoven or aggregated into rhizomorphic strands, branched, clamped; walls thin or irregularly slightly thickened, the hyphal walls echinulate with light microscopy, covered with nipple-shaped protuberances, as well as encrusted with prism-like crystals (up to 6 μm long) that are insoluble in KOH.

#### Chemical reactions.

(dried basidiomes): FeCl_3_ = positive, green-yellow; KOH = positive, cherry-red or pink; NH_4_OH = positive, golden-rod or vivid yellow; phenol = positive, light yellow; ethanol, FeSO_4_, and Melzer’s reagent = negative.

#### Known distribution and ecology.

NW China and SW China, and India. Gregarious habit on the ground in conifer or mixed conifer forests (e.g. *Abies* spp. and *Picea* spp.) at elevations ranging from 3000–3950 m.

#### Materials examined.

China. Gansu Province: Zhouqu Prefecture, under *Abies* spp., 6 August 2005, *X.T. Zhu 728* (HKAS 76577). Qinghai Province: Qilian mountains, 38°6.00'N, 100°7.03'E, alt. 3000 m, 21 August 2004, *H.A Wen 4305* (HMAS 132008); same location and date, *Q.B. Wang 438* (HMAS 97090). Sichuan Province: Seda Prefecture, *Picea*-*Juniperus* forests, 31°43.20'N, 100°43.17'E, alt. 3775–3925 m, 6 August 2005, *Z.W. Ge 783* (HKAS 49278); Litang Prefecture, 5 August 2007, *Z.W. Ge 1712* (HKAS 53797). Tibet: Linzhi City, 29°20.07'N, 094°18.00'E, alt. 3850 m, 19 July 2004, *Y.H. Wang 125* (HMAS 97248); Jilong Prefecture, on the ground in coniferous woods, 12 September 1990, *J.Y. Zhuang 3814* (HMAS 59867); Chengdu City, under forests dominated by *Picea* spp., 31°30.43'N, 097°20.07'E, alt. 3480–3550 m, 17 August 2004, *Z.W. Ge 381* (HKAS 46160); Riwoqe Prefecture, under *Picea* spp., 31°14.27'N, 096°31.92'E, alt. 3890 m, 12 August 2004, *Z.W. Ge 340* (HKAS 46120). Yunnan Province: Shangri-La Prefecture, Haba Snow Mountains, alt. 2800 m, 15 August 2008, *L.P. Tang 645* (HKAS 54876, ***Holotype***); Shangri-La Prefecture, 27°28.13'N, 099°25.03'E, alt. 3600 m, 15 August 2008, *T.Z. Wei* 172 (HMAS 250466).

#### Comments.

*Clavariadelphus amplus* is distinctive by its pink-orange to red-orange, bright basidiomes, obviously enlarged, truncate, depressed, sterile apices (up to 7.5 m diam.) at maturity, large basidiospores (8.2–11.0 × 5.1–6.4 μm), gregarious habit at high elevations, base mycelial hyphae with nipple-shaped protuberances and prism-like crystals and a cherry-red staining reaction to KOH. It is sold as an edible mushroom in markets in SW China. This taxon has a wide distribution in NW and SW China, including Gansu, Qinghai, Sichuan, Tibet and Yunnan Provinces. The data from GenBank (accession MT012805) also indicated its distribution of India.

This species was previously referred to as either *C. pallido-incarnatus* (Yuan and Sun 1995) or *C. truncatus* (Mao et al., 1993; Zang 1996; Mao 2009; Tang and Yang 2014; Tang 2015). *Clavariadelphus pallido-incarnatus*, a species described from the Pacific Northwest in North America, has pale pinkish-cinnamon basidiomes with fertile, non-truncated apices, no reactivity to KOH and habitat preference for coastal forests of *Sequoia sempervirens* and *Picea sitchensis* (Methven 1990). *Clavariadelphus truncatus* from Europe is readily confused with *C. amplus* as they have similar size and truncate sterile apex. However, *C. truncatus* has dark coloured basidiomes from yellow to cinnamon-brown or brown, narrower apices (up to 3.5 cm) and larger basidiospores (10.3–12.6 × 5.5–7.1 μm from neotype; Methven 1990). *Clavariadelphus unicolor* (Berk. & Ravenel) Corner, is also from North America and has enlarged sterile apices, but it is distinct in its reddish-brown to violet-brown basidiomes, narrow basidiospores with *Q*_m_ 2.1, a golden-yellow reaction to KOH and association with deciduous trees (Methven 1990).

So far, there are two species with sterile apices found in China, *C. amplus* and *C. gansuensis*. However, *C. gansuensis* has a narrower apex (up 1.6 cm), slightly broader basidiospores with a lower Q value (8.3–10.1 × 5.3–6.3 μm, *Q* = 1.47 –1.78, *Q*_m_ = 1.60) and a solitary growth habit. Except for the mentioned species, *C. amplus* is also similar to *C. pakistanicus*. *Clavariadelphus pakistanicus*, another species also from Asia, is distinct in smaller basidiomes (up to 12 cm high), with narrower fertile apices (up to 1.4 cm), smaller basidiospores (7.5–9.2 × 4.0–5.6 μm), solitary growth habit at lower elevations and violet-brown staining reactions to KOH (Hanif et al. 2014).

In the ITS tree, *C. amplus* exhibits a sister relationship with *C. pakistanicus* with strong support (Fig. 1).

### 3. Clavariadelphus elongatus

Taxon classificationFungiGomphalesClavariadelphaceae

J. Khan, Sher & Khalid, Phytotaxa 365: 184, 2018

28B51FE0-4946-5164-9141-99728F7D3743

**Figure 8.**
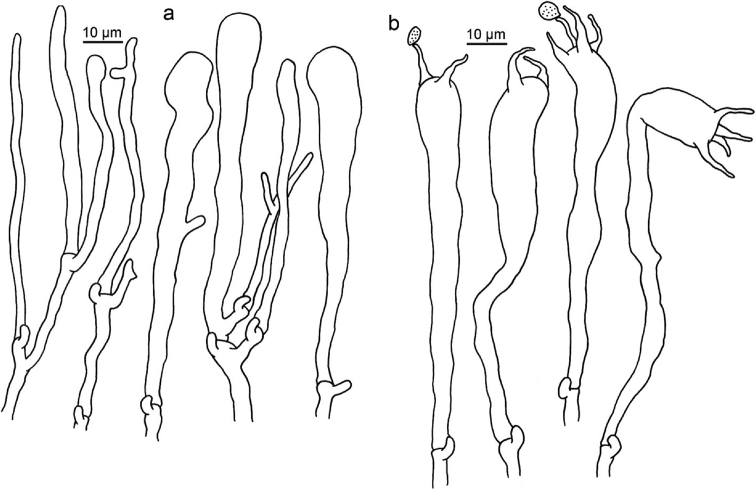
Microscopic features of *Clavariadelphus elongatus* (HKAS 76589). **a**Leptocystidia and immature basidia **b** Basidia.

Figs 2d , 2e , 3c , 4c , 5c , 8a , 8b

#### Note.

The following description is taken from Sher et al. (2018), field notes of the Chinese material including macro-morphology, growth habit, distribution, host plants and our examination of the specimens.

#### Description.

*Basidiomes* up to 28 cm high, 0.5–1.0 cm diam. basally, enlarged upwards to 1.5 cm diam., subcylindrical to fusiform, simple or occasionally branched, laterally compressed in age; *hymenium* longitudinally rugose, plum colour (13C2–4) or light purple to greyish-purple (14C2–3) or dull-lilac (15D2–3); *apex* tapered, subacute to obtuse, initially smooth, rugulose in age, caramel-brown to sandy-brown or sienna (6C5–6); *base* terete, smooth, white; *mycelial hyphae* scant, white; *flesh* initially solid, then soft and spongy in age. *Odour* and *taste* not recorded.

*Hymenium* extending over the apex of the basidiomata, composed of basidia and leptocystidia. *Basidia* 75–95 × 6–10 μm, clavate, hyaline, thin-walled, 4-spored, sterigmata 7–10 μm in length. *Basidiospores* [40/2/2] (8.3–) 9.0–11.0 (–12.0) × (5.5–) 5.7–7.4 μm, *Q* = (1.43–) 1.44–2.04 (–2.31), *Q*_m_ = 1.71 ± 0.16, narrowly ellipsoid to ellipsoid, ovate or amygdaliform, with a small apiculus, inamyloid, thin-walled, hyaline in KOH, smooth. *Leptocystidia* 70–75 × 3.5–4.5 μm, scattered amongst and scarcely projecting beyond the basidia, cylindrical to narrowly clavate, thin-walled, smooth, hyaline, non-pigmented, clamped, inflated apically at maturity, at times with apical or subapical branches. *Mycelial hyphae* 2–3 or 6–8 μm diam., interwoven or aggregated into rhizomorphic strands, branched, clamped; the hyphal walls echinulate with light microscopy, encrusted with massive triangular or irregular, flaky crystals up 1 μm high, which are insoluble in KOH.

#### Chemical reactions.

(dried basidiomes): KOH = positive, light yellow; FeCl_3_ = positive, green-yellow; NH_4_OH = positive, orange; ethanol, FeSO_4_, phenol and Melzer’s reagent = negative.

#### Known distribution and ecology.

NW and SW China (in this study), Pakistan (Sher et al. 2018). Solitary to scattered on the ground in coniferous woods (*Abies* spp. and *Picea* spp.) or mixed with broad-leaved trees (*Quercus* spp., *Rhododendron* spp. and *Salix* spp.) at elevations ranging from 3000–4350 m.

#### Materials examined.

China. Gansu Province: Zhouqu Prefecture, Shatan National Forest Park, *Abies* spp. woods, 16 August 2012, *X.T Zhu 740* (HKAS 76589). Sichuan Province: Litang Prefecture, Gaowa, Kobresia-Bistorta meadows with extensive areas of dwarf *Rhododendron* and *Salix* scrub with *Picea* spp., 30°10.10'N, 100°35.12'E, alt. 4300–4350 m, 8 August 2006, *Z.W. Ge 1221* (HKAS 50801); Yajiang Prefecture, meadows with shrub thickets and *Picea* spp. forests, 30°2.67'N, 101°18.48'E, alt. 3850–3870 m, 4 August 2006, *Z.W. Ge 1162* (HKAS 50742). Yunnan Province: Yulong Prefecture, Lizui Village, mixed coniferous and broad-leaved forests of *Picea* spp. and *Quercus* spp., alt. 3000 m, 23 August 2007, *Y. Zhang 36* (HKAS 52425); Shangri-La Prefecture, 27°29.00'N, 99°25.00'E, alt. 3600 m, 13 August 2008, *T.Z. Wei 150* (HMAS 260746).

#### Comments.

*Clavariadelphus elongatus* was originally described from Pakistan (Sher et al. 2018). In this study, it was found in NW and SW China. This species is unique in its greyish-purple basidiomes with acute to subacute, non-enlarged apex, hyphae of the basal mycelium encrusted with massive, flaky crystals and basidiomes having a light yellow reaction to KOH. *Clavariadelphus himalayensis*, another Asian taxon, might be confused with *C. elongatus* since both have a tinge of grey-purple when young. However, *C. himalayensis* is distinct in having smaller basidiomes, pastel-red colouration at maturation, shorter basidiospores (8.2–9.4 × 5.0–5.5 μm), hyphae of the basal mycelium covered nipple-shaped protuberances without crystals and basidiomes having a brown-yellow reaction to KOH.

Phylogenetically, *C. elongatus* is related to *C. pistillaris* and the sequence of “*C. occidentalis*” from GenBank with weak support (Fig. 1).

### 4. Clavariadelphus gansuensis

Taxon classificationFungiGomphalesClavariadelphaceae

J. Zhao & L.P. Tang sp. nov.

2C3EA552-C1E1-514E-B95C-9AFC1A002716

MycoBank No: 830272

**Figure 9.**
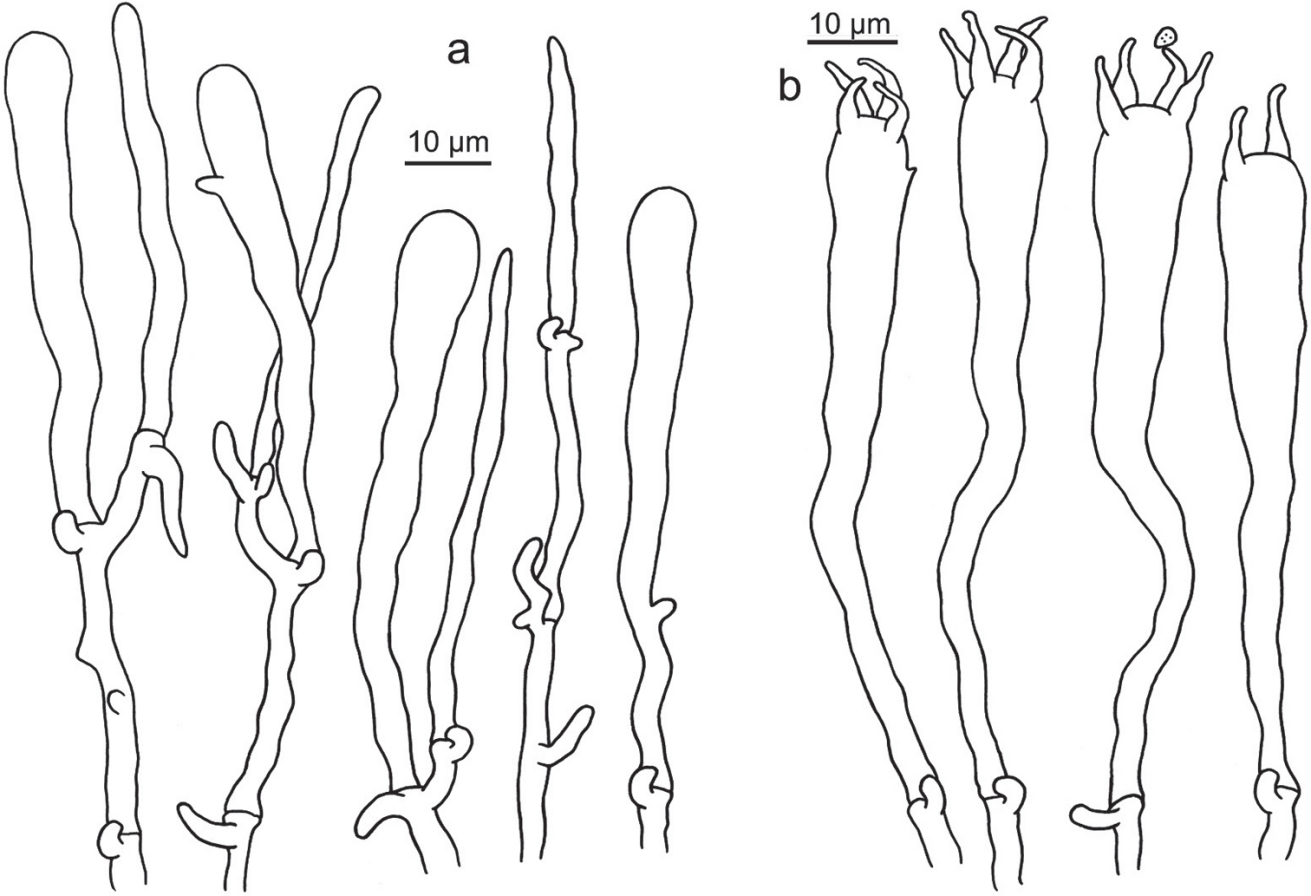
Microscopic features of *Clavariadelphus gansuensis* (HKAS 76487, holotype). **a**Leptocystidia and immature basidia **b** Basidia.

Figs 2f , 3d , 4d , 5d , 9a, b

#### Diagnosis.

This species is characterised by its orange, clavate basidiomes with slightly enlarged, truncate, sterile apex, broadly ellipsoid to ellipsoid basidiospores, hyphae of the basal mycelium with nipple-shaped protuberances and prism-like crystals and basidiomes that turn pink or light cherry-red in KOH. It differs from *C. truncatus* by the latter’s robust, darker basidiomes with enlarged apices, and larger basidiospores.

#### Etymology.

Latin “*gansuensis*” refers to the holotype location in Gansu Province.

#### Description.

*Basidiomes* up to 9 cm high, enlarged upwards to 1.6 cm diam., simple, clavate; *hymenium* longitudinally rugose, pruinose, light yellow to greyish-orange at maturity; *apex* initially obtuse or broadly rounded, flattening laterally, then truncate, slightly rugose, light orange or melon-orange (5A5–7) to orange (6A6–7) in age; *base* terete, smooth, pruinose, dirty white or pallid where covered, otherwise pruinose, pale orange or light orange (5A3–4); *mycelial hyphae* white; *flesh* initially solid, then soft and spongy upwards as the apex enlarges, white to pallid. *Odour* and *taste* not recorded.

*Hymenium* limited to the side of basidiomata, composed of basidia and leptocystidia; the apex of basidiomata composed of sterile elements 15–25 × 5–7 μm, clavate, thin-walled, smooth, clamped. *Basidia* 75–90 × 8–10 μm, clavate, hyaline, thin-walled to thick-walled, 4-spored, sterigmata 7–10 μm in length. *Basidiospores* [20/1/1] 8.3–10.1 (–10.3) × 5.3–6.3 (–6.4) μm, *Q* = (1.34–) 1.47 –1.78 (–1.83), *Q*_m_ = 1.60 ± 0.09, ellipsoid to broadly ellipsoid, ovate or amygdaliform, with a small apiculus, inamyloid, thin-walled, hyaline in KOH. *Leptocystidia* 50–65 × 3–5 μm, scattered amongst and scarcely projecting beyond the basidia, cylindrical to narrowly clavate, thin-walled, smooth, hyaline, non-pigmented, clamped, inflated apically at maturity, at times with apical or sub-apical branches. *Mycelial hyphae* 2–3 μm diam., interwoven or aggregated into rhizomorphic strands, branched, clamped; the hyphal walls echinulate with light microscopy, covered with massive nipple-shaped protuberances, as well as encrusted with prism-like crystals up 5 μm long that are insoluble in KOH.

#### Chemical reactions.

(dried basidiomes): KOH = positive, pink, light coral or light cherry-red; FeCl_3_ = positive, green-yellow; NH_4_OH = positive, golden-rod or vivid yellow; phenol = positive, yellow; ethanol, FeSO_4_ and Melzer’s reagent = negative.

#### Known distribution and ecology.

NW China, Gansu Province. Solitary on the ground in coniferous woods (*Abies* spp.) or mixed with broad-leaved trees (*Betula* spp. and Rosaceae) at elevations of approximately 3000 m.

#### Materials examined.

China. Gansu Province: Lintan Prefecture, Yeliguan National Forest Park, coniferous woods (*Abies* spp.) or mixed with *Betula* spp. and Rosaceae plants, alt. 3000 m, 10 August 2012, *X.T. Zhu 638* (HKAS 76487, ***Holotype***); Wudu Prefecture, September 1992, *M.L. Tian M6465* (HMAS 63052).

#### Comments.

*Clavariadelphus gansuensis*, currently known only from NW China, is distinct by its slender, clavate, orange basidiomes with truncate apex, ellipsoid basidiospores (8.3–10.1 × 5.3–6.3 μm), pink staining reaction to KOH, hyphae of the basal mycelium with nipple-shaped protuberances and prism-like crystals and solitary growth habit in coniferous or mixed forests.

This species is most likely to be confused with several taxa, including *C. amplus*, *C. pallido-incarnatus*, *C. pakistanicus*, *C. truncatus* and *C. unicolor*. The comparison between *C. gansuensis* and *C. amplus* can be found in our treatment of *C. amplus*.

According to our phylogenetic analyses, *C. gansuensis* is allied with the sequence of “*C. truncatus*” from GenBank with strong support (Fig. 1).

### 5. Clavariadelphus himalayensis

Taxon classificationFungiGomphalesClavariadelphaceae

Methven, Mem. New York Bot. Garden 49: 152, 1989

A3360663-90F3-5FCB-8AF8-A2A7D37BA9BA

**Figure 10.**
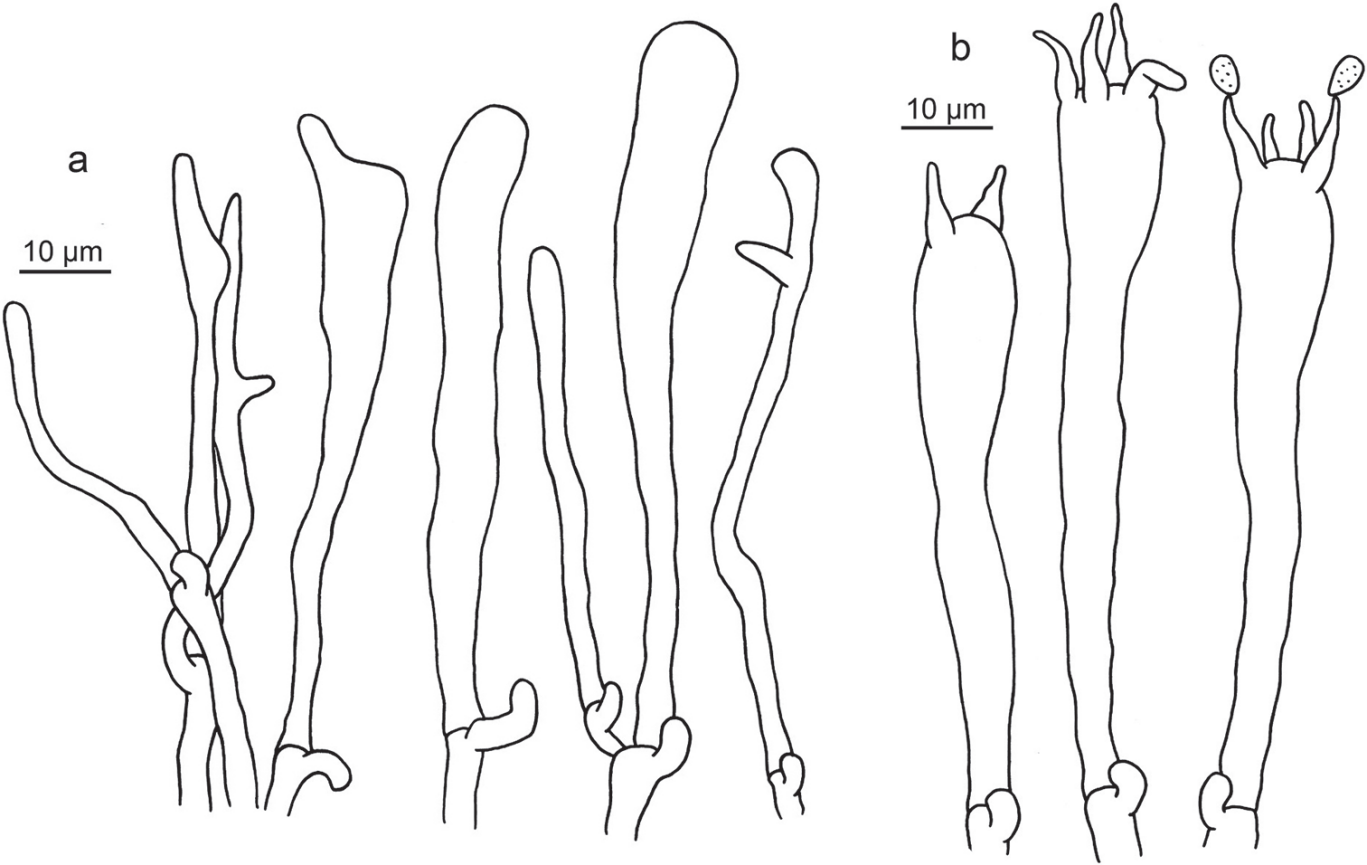
Microscopic features of *Clavariadelphus himalayensis* (HKAS 58811). **a**Leptocystidia and immature basidia **b** Basidia.

Figs 2g , 3e , 4e, f , 5e , 10a, b

#### Note.

The following description is mainly from Methven (1989), combined with our field notes, including macro-morphology, growth habit, distribution, host plants and examination.

#### Description.

*Basidiomes* up to 15 cm high, 1–1.5 cm diam. basally, slightly enlarged towards to 2 cm diam., simple, narrow clavate, ligulate to spathulate, laterally compressed in mature specimens; *hymenium* initially smooth, longitudinally rugose in age, greyish-red to pastel-red; *apex* obtuse, smooth, concolorous with the hymenium; surface not staining where cut or bruised; *base* terete, smooth, pruinose, pallid-white; *mycelial hyphae* interwoven, white to pallid; *flesh* soft and spongy, hollow apically in age, white to cream colour, not staining on exposure. *Odour* and *taste* not recorded.

*Hymenium* extending over the apex of basidiomata, composed of basidia and leptocystidia. *Basidia* 75–95 × 8–11 μm, clavate, hyaline, thin-walled, (2–) 4-spored, sterigmata 8–10 μm in length. *Basidiospores* [20/1/1] (7.8–) 8.2– 9.4 (–9.6) × (4.6–) 5.0–5.5 (–6.0) μm, *Q* = 1.50–1.82 (–1.90), *Q*_m_ = 1.56 ± 0.08, ellipsoid to broadly ellipsoid or ovate, with a small apiculus, inamyloid, thin-walled, hyaline in KOH, smooth. *Leptocystidia* 50–70 × 2.5–3.5 μm, scattered amongst and scarcely projecting beyond the basidia, cylindrical to narrowly clavate, thin-walled, smooth, hyaline, non-pigmented, clamped, inflated apically at maturity, at times with apical or subapical branches. *Mycelial hyphae* 1–2 or 3–5 μm diam., interwoven or aggregated into rhizomorphic strands, branched, clamped; walls thin or irregularly slightly thickened, the hyphal walls echinulate under light microscopy, covered nipple-shaped protuberances with SEM.

#### Chemical reactions.

(dried basidiomes): KOH = positive, golden-yellow; FeCl_3_ = positive, green-yellow; NH_4_OH = positive, orange; ethanol, FeSO_4_, Melzer’s reagent and phenol = negative.

#### Known distribution and ecology.

SW China (in this study) and India (Methven 1989). Solitary to gregarious habit on the ground in mixed woods at elevations above 3200 m.

#### Materials examined.

China. Yunnan Province: Shangri-La Prefecture, mixed coniferous (*Pinus* spp.) and broad-leaved forests (*Caragana* spp., dwarf *Quercus monimotricha* and *Sanguisorba* spp.), 27°28.55'N, 99°53.05'E, alt. 3280 m, 27 June 2006, *Z.W. Ge 1113* (HKAS 50684). Lijiang Prefecture, mixed conifers, alt. 3300 m, 27 August 2009, *Q. Cai 146* (HKAS 58811).

#### Comments.

*Clavariadelphus himalayensis* was originally described from India (Methven 1989). It is the first report from China. Chinese collections match the original descriptions except for slightly smaller basidiospores (8.2–9.4 × 5.0–5.5 μm). The difference in basidiospore size might be from measurement error or the collections being from different geographical regions. *Clavariadelphus himalayensis* is distinct by its pastel-red to greyish-red, ligulate to spathulate basidiomes flesh that does not stain where bruised or cut, broadly ellipsoid basidiospores (9–11 × 5–6 μm from the holotype; Methven 1989), hyphae of the basal mycelium with nipple-shaped protuberances and a negative reaction with phenol. Other taxa from Asia, which might be confused with *C. himalayensis* include *C. mirus* (Pat.) Corner and *C. yunnanensis*. Although similar in size to those of *C. himalayensis*, the basidiomes of *C. mirus* are light brown to brown and produce broadly ovate, larger basidiospores (10–13 × 6–8 μm; Methven 1990). *Clavariadelphus yunnanensis*, known from northern India and SW China, is distinct by its larger basidiomes that are light brown, larger basidiospores (10–13.5 × 6.5–8 μm), hyphae of the basal mycelium covered by massive nipple-shaped protuberances and a light yellow staining reaction with phenol. Additionally, the flesh of *C. himalayensis* does not stain where bruised or cut, whereas the flesh of *C. mirus* and *C. yunnanensis* slowly stains brunnescent to russet on exposure.

The phylogenetic analyses show that *C. himalayensis* is allied with the sequence of “*C. pistillaris*” and *Clavariadelphus* (JQ991679 from Zhejiang Province, China) from GenBank with weak support (Fig. 1). More data are needed for understanding the phylogenetic relationship of the three species.

### 6. Clavariadelphus khinganensis

Taxon classificationFungiGomphalesClavariadelphaceae

J. Zhao, L.P. Tang & P. Zhang sp. nov.

14004616-12B2-5647-B537-B3A48FAB573D

MycoBank No: 830273

**Figure 11.**
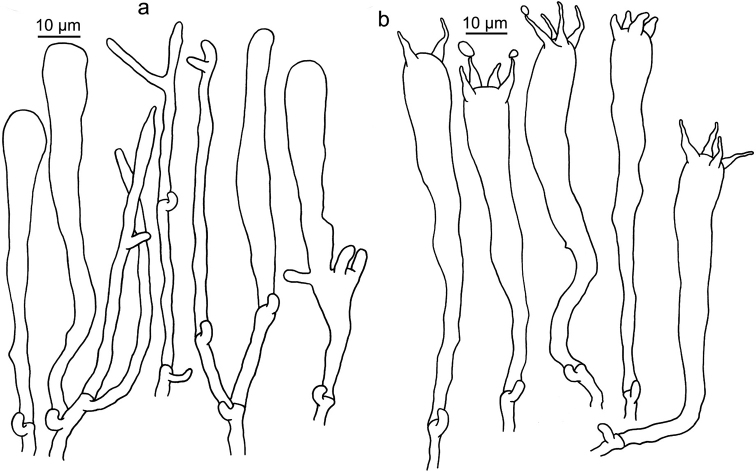
Microscopic features of *Clavariadelphus khinganensis* (MHHNU 7789, holotype). **a**Leptocystidia and immature basidia **b** Basidia.

Figs 2h–i , 3f , 4g , 11a, b

#### Diagnosis.

This species is distinct from other taxa in *Clavariadelphus* by the yellowish-brown, clavate basidiomes with slightly enlarged apex, narrowly ellipsoid basidiospores and basidiomes that turn very light yellow in KOH.

#### Etymology.

Latin “*khinganensis*” refers to the holotype location, Greater Khingan Mountains or Da Xing’an Ling, in NE China.

#### Description.

*Basidiomes* up to 12.5 cm high, around 0.8 cm diam. basally, 2.5 cm diam. apically, simple, initially subcylindrical to subfusiform, enlarged upwards in age, then clavate to broadly clavate, finally irregularly laterally compressed; *hymenium* initially smooth, longitudinally rugose to rugulose in age, pale yellow-brown (4A3) or pale orange (5A 4–6) to greyish-orange (5B4–5, 6B4–5); *apex* obtuse or broadly rounded, rugose, concolorous with the hymenium at maturity; *base* terete, smooth, white to pallid when covered, otherwise pale yellow (4A4–5) to light orange (5A4–6); *mycelial hyphae* interwoven, white; *flesh* initially solid, becoming soft and spongy upwards as the apex enlarges in age, dirty white. *Odour* and *taste* not recorded. *Spore deposit* not recorded.

*Hymenium* extending over the apex of basidiomata, composed of basidia and leptocystidia. *Basidia* 85–105 × 8–11 μm, clavate, hyaline, thin-walled, 4-spored, sterigmata 9–10 μm in length. *Basidiospores* [20/1/1] 9.2–12.0 × 4.6–6 μm, *Q* = 1.6–2.2, *Q*_m_ = 1.97 ± 0.17, narrowly ellipsoid or amygdaliform, with a small apiculus, inamyloid, thin-walled, hyaline in KOH, smooth. *Leptocystidia* 60–70 × 3–4 μm, scattered amongst and scarcely projecting beyond the basidia, cylindrical to narrowly clavate, thin-walled, smooth, hyaline, non-pigmented, clamped, inflated apically at maturity, at times with apical or subapical branches. *Mycelial hyphae* lacking material.

#### Chemical reactions.

(dried basidiomes): KOH = positive, very light yellow; ethanol, FeCl_3_, FeSO_4_, phenol, Melzer’s reagent and NH_4_OH = negative.

#### Known distribution and ecology.

N China. Solitary on the ground in broad-leaved forests at around 800 m altitude.

#### Materials examined.

CHINA. Jilin Province: Antu Prefecture, Er-dao-bai-he Town, Changbai Mountains, mainly broad-leaved forests (*Betula platyphylla*, *Corylus mandshurica*, and *Quercus monimotricha*), mixed with the coniferous tree (*Pinus koraiensis*), 42°24.05'N, 128°6.00'E, alt. 753 m, 18 August 2019, *H.Y. Huang 368* (MHKMU H.Y. Huang 368). Inner Mongolia: De-er-bu-er Town, Greater Khingan Mountains, alt. 800 m, 6 August 2013, *P. Zhang 1289* (MHHNU 7789 ***Holotype***); Ku-ti-he Town, Zha-lan-tun City, 24 July 1985, *W. Huang s. n.* (HMAS 49920).

#### Comments.

*Clavariadelphus khinganensis*, known from broad-leaved forests in N China, is distinct by its solitary habit at low elevations (around 800 m), small size, pale brown-orange basidiomes, ellipsoid basidiospores and very pale yellow reaction in KOH.

Morphologically, *C. khinganensis* is quite similar to two Asian taxa, *C. mirus* and *C. yunnanensis*. However, *C. mirus* was originally described from northern Vietnam and has larger basidiomes, broader basidiospores and a tropical distribution (Butan, India, Nepal; Methven 1990). *Clavariadelphus yunnanensis* is unique in its habit, growing with conifers at high elevations (above 3000 m), has darker colouration and larger basidiomes (up to 20 cm high), broader basidiospores and basidiomes with yellow reactivity in KOH.

Interestingly, *C. khinganensis* is clustered with a collection labeled as “*C. truncatus*” from Canada, the GenBank accession DQ097871 (Durall et al. 2006) and there are no genetic differences on ITS (Fig. 1). It indicates *C. khinganensis* may be distributed in Canada. More data from North America are needed to confirm the distribution pattern of this species. The sister relationship of *C. khinganensis* cannot be resolved according to the present data.

### 7. Clavariadelphus ligula

Taxon classificationFungiGomphalesClavariadelphaceae

(Schaeff.) Donk, Rev. Niederl. Homob. Aphyll. 2: 73, 1933

D65AE4D8-241D-5801-A5A8-2DFC3E9DA46B

Figs 3g , 12a, b

#### Note.

The following taxonomic description is drawn from Methven (1990) and our observations.

**Figure 12.**
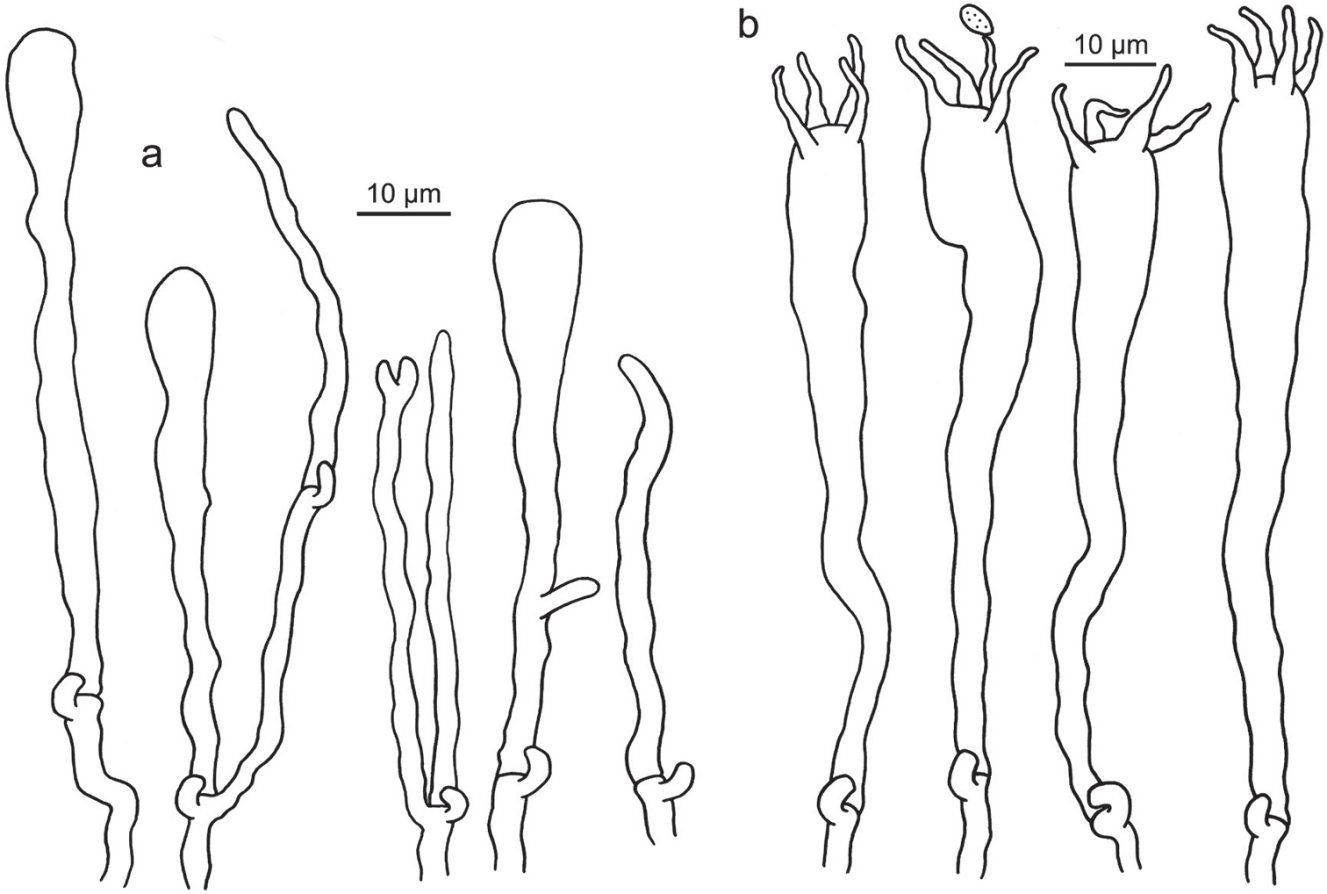
Microscopic features of *Clavariadelphus ligula* (HMAS 35954). **a**Leptocystidia and immature basidia **b** Basidia.

#### Description.

*Basidiomes* up to 10 cm high, 0.2–0.8 cm diam. basally, slightly enlarged upwards, simple, narrowly clavate to clavate; *hymenium* longitudinally rugose in age, light yellow, brownish-orange to light brown at maturity; *apex* subacute to obtuse or broadly rounded, surface slightly rugulose, concolorous with the hymenium; surface slowly staining brownish-orange to brownish-grey where cut or bruised; *base* terete, initially pale yellow to light yellow, then brownish-orange to light brown to brown; *mycelial hyphae* white to pallid; *flesh* initially solid, becoming soft and spongy upwards as the apex enlarges in age, white to pallid. *Odour* not distinctive. *Taste* not distinctive or slightly sweet. *Spore deposit* yellowish-white to light buff in mass.

*Hymenium* extending over the apex of basidiomata, composed of basidia and leptocystidia. *Basidia* 45–85 × 8–11 μm, clavate, hyaline, thin-walled, 4-spored, sterigmata 9–10 μm in length. *Basidiospores* 11.0–14.0 × 4.0–5.5 μm, *Q* = 2.4–3.1, *Q*_m_ = 2.7, narrowly ellipsoid, with a small apiculus, inamyloid, thin-walled, hyaline in KOH, smooth. *Leptocystidia* 40–80 × 2.5–5 μm, scattered amongst and scarcely projecting beyond the basidia, cylindrical to narrowly clavate, thin-walled, smooth, hyaline, non-pigmented, clamped, inflated apically at maturity, at times with apical or subapical branches. *Mycelial hyphae* 2–4 μm diam., interwoven or aggregated into rhizomorphic strands, branched, clamped. Insufficient material to perform SEM.

#### Chemical reactions.

(dried basidiomes): KOH = positive, lemon-chiffon; NH_4_OH = positive, orange; ethanol, FeCl_3_, FeSO_4_, Melzer’s reagent and phenol = negative.

#### Known distribution and ecology.

Widespread in the Northern Hemisphere, including in North America, Bulgaria, NE China, England, Estonia, Finland, Germany, India, Italy, Sweden and Switzerland (Methven 1990). Scattered to gregarious habit on the ground in mixed woods (*Abies*, *Picea*, *Pinus*, *Thuja* and *Tsuga*).

#### Materials examined.

China. Heilongjiang Province: Linkou Prefecture, 19 August 1972, *X.L. Mao*, *s. n.* (HKAS 35954); same location, *Q.X. Wu*, *s. n.* (HMAS 51688). Czech: 2 September 1960, *M. Geesteranus 13290* (HMAS 41146).

#### Comments.

*Clavariadelphus ligula* was originally described from Germany, but was also reported in China (Mao 2009). Our study confirms that this species is mainly found in N China, whereas our data do not support the previous report of the distribution in SW China (Mao et al. 1993; Mao 2009). The basidiospores of Chinese collections (11.0–14.0 × 4.0–5.5 μm, *Q* = 2.4–3.1, *Q*_m_ = 2.7) are smaller and broader than the neotype of *C. ligula* from Germany (12.0–16.5 × 3.5–4.5 μm, *Q* = 2.9–4.6, *Q*_m_ = 3.7; Methven 1990).

Morphologically, *C. ligula* and *C. sachalinensis* are similar in the field. However, *C. sachalinensis* has more elongated, narrower basidiospores (21–24 × 4–6 μm, *Q* = 3.5–5.0, *Q*_m_ = 4.2). Additionally, *C. ligula* lacks any reaction with FeCl_3_, whereas *C. sachalinensis* turns green-yellow in FeCl_3_. *Clavariadelphus yunnanensis* is likely to be confused with *C. ligula* when young. However, *C. yunnanensis* differs in that it has larger basidiomes (up to 20 cm high), smaller and broader basidiospores (9.0–11.0 × 4.6–6.4 μm, *Q* = 1.32–1.72, *Q*_m_ = 1.56) and a positive reaction with phenol.

The phylogenetic analyses show that *C. ligula* is allied with the sequence of *C. americanus* from GenBank with strong support (Fig. 1).

### 8. Clavariadelphus sachalinensis

Taxon classificationFungiGomphalesClavariadelphaceae

(S. Imai) Corner, Ann. Bot. Mem. I: 282, 1950

5C466E1A-D6B7-58BD-84ED-B2E3BE78B16A

**Figure 13.**
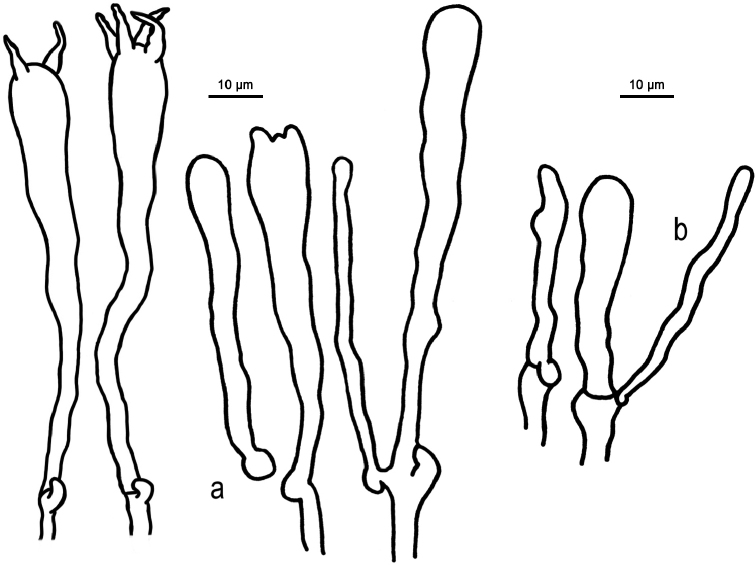
Microscopic features of *Clavariadelphus sachalinensis* (MHHNU 7816). **a** Basidia **b**Leptocystidia and immature basidia.

Figs 2j , 3h , 5f, g , 13a, b

#### Note.

The following taxonomic description is drawn from Methven (1989), combined with our field notes, including macro-morphology, growth habit, distribution, host plants and observations.

#### Description.

*Basidiomes* up to 8 cm high, 0.3–0.6 cm diam. basally, slightly enlarged upwards 0.8–1.2 cm diam., simple, initially cylindrical to subcylindrical, then narrowly clavate to clavate; *hymenium* longitudinally rugose in age, tawny or light walnut-brown to light brown at maturity; *apex* subacute, obtuse to broadly rounded, surface smooth to slightly rugulose, concolorous with the hymenium; surface slowly staining, brown or dark brown where cut or bruised, staining more conspicuously; *base* terete, pubescent to tomentose, initially pale yellow to light yellow, then brownish-orange to light brown; *mycelial hyphae* greyish to pallid; *flesh* initially solid, becoming soft and spongy upwards, white to pallid, staining on exposure. *Odour* and *taste* not distinctive. *Spore deposit* yellowish-white to light buff.

*Hymenium* extending over the apex of basidiomata, composed of basidia and leptocystidia. *Basidia* 65–105 × 8–12.5 μm, clavate, hyaline, thin-walled, (2–) 4-spored, sterigmata 8–10 μm in length. *Basidiospores* 21–24 × 4–6 μm, *Q* = 3.5–5.0, *Q*_m_ = 4.2, narrowly ellipsoid, boletoid or sway-backed in profile, with a small apiculus, inamyloid, thin-walled, hyaline in KOH, smooth. *Leptocystidia* 50–70 × 2.5–5 μm, scattered amongst and scarcely projecting beyond the basidia, cylindrical to narrowly clavate, thin-walled, smooth, hyaline, non-pigmented, clamped, inflated apically at maturity, at times with apical or subapical branches. *Mycelial hyphae* 2–8 μm diam., interwoven or aggregated into rhizomorphic strands, branched, clamped; hyphal walls smooth with light microscopy and SEM.

#### Chemical reactions.

(dried basidiomes): KOH = positive, light yellow; FeCl_3_ = positive, green-yellow; NH_4_OH = positive, orange; ethanol, phenol, FeSO_4_ and Melzer’s reagent = negative.

#### Known distribution and ecology.

N China (in this study) and SW China (Methven 1990). Gregarious habit on fallen needles and other debris under conifers, especially pine at elevations ranging from 2000–3600 m.

#### Materials examined.

China. Inner Mongolia: Mo er dao ga National Forests, Great Khingan Mountains, 8 August 2013, *P. Zhang 1316* (MHHNU 7816). Sichuan Province: Hongyuan Prefecture, Kangle Town, alt. 3600 m, 14 August 1998, *M.S. Yuan 3361* (HKAS 33844).

#### Comments.

*Clavariadelphus sachalinensis* was proposed by Imai, based on Japanese collections as a species of *Clavaria* and then transferred to genus *Clavariadelphus* (Imai 1930, Corner 1950). In China, *C. sachalinensis* was previously reported with distribution in SW China (Methven 1990; Zang 1996) and is also found in northern regions of China. *Clavariadelphus sachalinensis* is similar to *C. ligula* and *C. yunnanensis*. Their differences are described in our discussion of *C. ligula*.

### 9. Clavariadelphus yunnanensis

Taxon classificationFungiGomphalesClavariadelphaceae

Methven, Mem. New York Bot. Garden 49: 156 1989

C2A9164A-E9D8-5AB7-8859-12FFA6140A22

**Figure 14.**
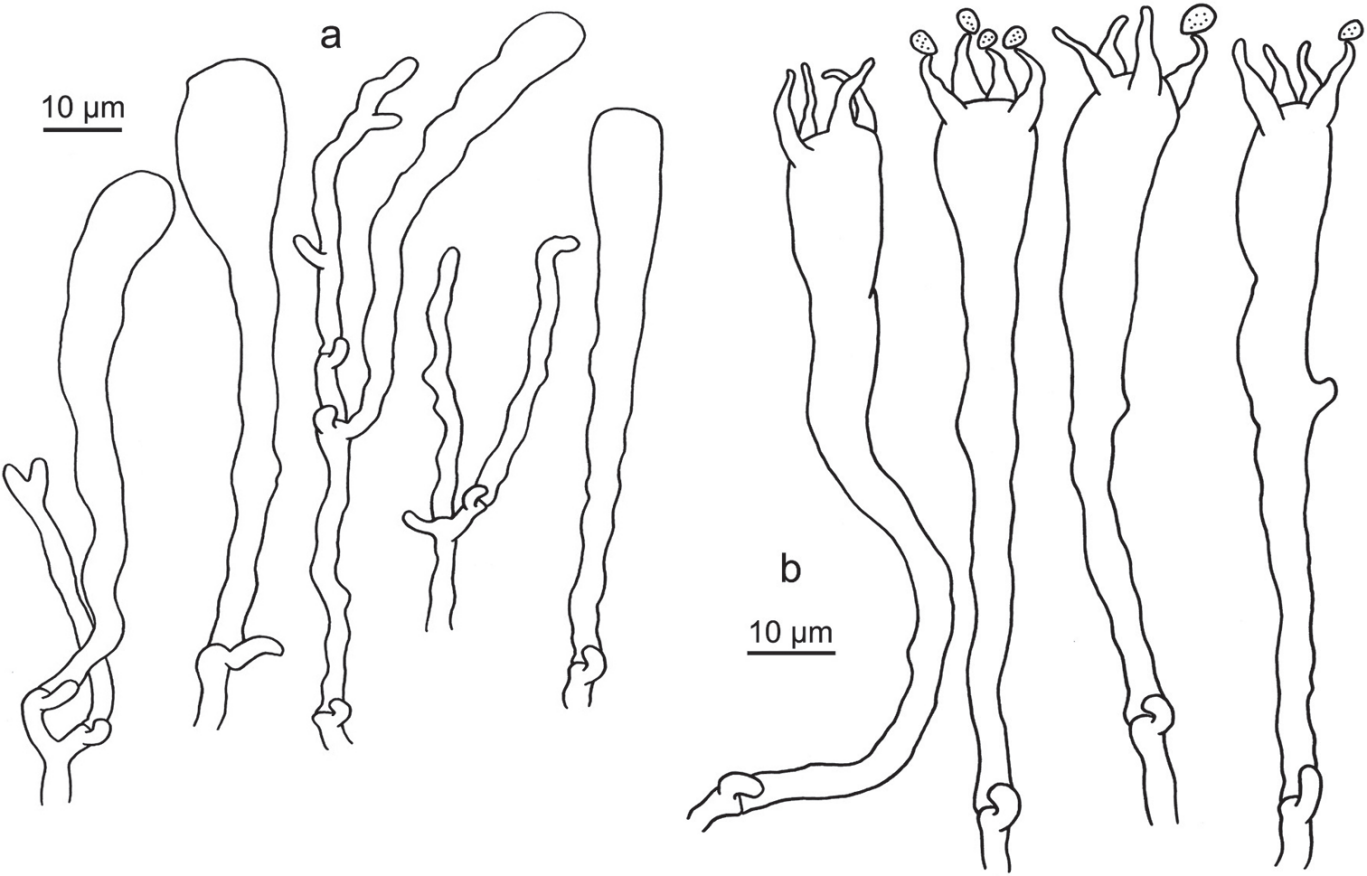
Microscopic features of *Clavariadelphus yunnanensis* (HKAS 57731). **a**Leptocystidia and immature basidia **b** Basidia.

Figs 2j–l , 3i , 4h , 5h , 14a, b

#### Note.

The following taxonomic description is mainly drawn from Methven (1989). Field notes including macro-morphology, growth habit, distribution and host plants, SEM characteristics and chemical tests are from this study.

#### Description.

*Basidiomes* up to 20 cm high, 0.5 cm diam. basally, enlarged upwards 2 cm diam., simple, initially cylindrical to subcylindrical, then narrowly clavate, subolanceolate; *hymenium* initially smooth, longitudinally rugose to rugulose in age, light brown to cinnamon at maturity; *apex* obtuse, smooth to rugose, concolorous with the hymenium; surface slowly staining, russet to umber; *base* terete, smooth, pale cinnamon or pale ochraceous-buff; *mycelial hyphae* white; *flesh* initially solid, becoming soft and spongy upwards as the apex enlarges, white to pinkish-buff. *Odour* not distinctive. *Taste* slightly bitter. *Spore deposit* white.

*Hymenium* extending over the apex of the basidiomata, composed of basidia and leptocystidia. *Basidia* 70–80 × 8–9 μm, clavate, hyaline, thin-walled, (2–) 4-spored, sterigmata 7–10 μm in length. *Basidiospores* [40/2/2] (8.8–) 9.0–11.0 × 4.6–6.4 (–7.4) μm, *Q* = (1.29–) 1.32–1.72 (–1.76), *Q*_m_ = 1.56 ± 0.11, ellipsoid to broadly ellipsoid, ovate or amygdaliform, smooth. *Leptocystidia* 40–60 × 2.5–3.5 μm, scattered amongst and scarcely projecting beyond the basidia, cylindrical to narrowly clavate, thin-walled, smooth, hyaline, non-pigmented, clamped, inflated apically at maturity, at times with apical or subapical branches. *Mycelial hyphae* 2–4 μm diam., parallel, interwoven or aggregated into rhizomorphic strands, branched, clamped; walls thin or irregularly slightly thickened, the hyphal walls echinulate with light microscopy, covered with massive nipple-shaped protuberances and lacking crystals with SEM.

#### Chemical reactions.

(dried basidiomes): KOH = positive, golden-yellow; FeCl_3_ = positive, green-yellow; NH_4_OH = positive, golden-rod or vivid yellow; phenol = positive, light yellow; ethanol, FeSO_4_ and Melzer’s reagent = negative.

#### Known distribution and ecology.

SW China and northern India (Methven 1989). Either solitary, scattered or gregarious habit on the ground in mixed deciduous-coniferous forests in association with several genera (e.g. *Abies*, *Berberis*, *Picea*, *Pinus*, *Quercus*, *Rosa* and *Salix*) at elevations ranging from 2200–3600 m.

#### Materials examined.

China. Sichuan Province: Hongyuan Prefecture, Shuajing Temple, *Picea*, alt. 3400 m, 3 August 1996, *M.S. Yuan 2375* (HKAS 30752); Kangding Prefecture, Liuba, alt. 3500 m, 9 September 1996, *M.S. Yuan 2686* (HKAS 31136); Kangding Prefecture, Zheduo Mountains, shrubs dominated by *Berberis*, *Quercus*, *Rosa*, *Salix*, alt. 3585 m, 14 August 2008, *Z.W. Ge 903* (HKAS 49398). Yunnan Province: Shangri-La Prefecture, 19 August 2008, 28°18.00'N, 98°33.00'E, alt. 3100 m, *T.Z. Wei 270* (HMAS 250510); Deqing Prefecture, Xiaruo, 18 September 2010, *HBB2010-D15* (HKAS 62644); Jianchuan Prefecture, Shibao Mountains, 14 August 2003, *Z.W. Ge 4* (HKAS 43816); same location, 30 August 2009, *G. Wu 199* (HKAS 57731); Kunming City, Yeya Lake, alt. 2200 m, 22 September 2012, *Z.L. Yang 5629* (HKAS 77288); Shangri-La Prefecture, Haba Mountains, 13 August 2008, *L.P. Tang 618* (HKAS 54849); Shangri-La Prefecture, 16 August 2008, *T.Z. Wei 271* (HMAS 250466); Shangri-La Prefecture, Bita Lake, 24 August 2009, *Q. Cai 122* (HKAS 58789); same location and date, *G. Wu 127* (HKAS 57659); Weixi Prefecture, Qizong, 19 September 2010, *HBB2010-W21* (HKAS 61417); Yulong Prefecture, Yulong Snow Mountains, Sandaowan, under *Abies* spp., alt. 3200 m, 1 August 1995, *M. Zang 12514* (HKAS 30038); Yulong Prefecture, Lizui Village, 20 August 2008, *Q. Zhao 8262* (HKAS 55244); Yulong Prefecture, Jiuhe, 20 August 2010, *G. Wu 327* (HKAS 63558); Yulong Prefecture, Yulong Snow Mountains, Ganhaizi, under *Picea* spp., alt. 3100 m, 3 September 1986, *M. Zang 10739* (HKAS 17788); Yulong Prefecture, Yulong Snow Mountains, Yu Lake, *Abies* forests, alt. 3000 m, 1 August 1985, *M. Zang 10220* (HKAS 15063); Yulong Prefecture, Yulong Snow Mountains, 6 September 1986, *R.H. Petersen s. n.* (HKAS 20067); same location and date, *R.H. Petersen s. n.* (HKAS 20068); Yulong Prefecture, Wenhai, 17 September 2012, *G. Wu 1054* (HKAS 77226).

#### Comments.

*Clavariadelphus yunnanensis* is quite common in SW China where it was previously reported as *C. ligula* or *C. pistillaris* (Mao et al. 1993; Yuan and Sun 1995; Zang 1996; Mao 2009). It is well characterised by its cinnamon buff, large basidiomes, broadly ellipsoid basidiospores, hyphae of the basal mycelium covered with nipple-shaped protuberances and occurrence at high elevation forests. This taxon is also similar to *C. ligula* and *C. sachalinensis*, but differs microscopically in the size and shape of the basidiospores (see the comments under *C. ligula*). Immature fruit bodies of *C. yunnanensis* are similar to *C. griseoclavus*. However, the latter can be distinguished from smaller basidiomata (less than 13 cm high), narrower apex (less than 1.5 cm diam.) and narrower basidiospores (*Q*_m_ 1.89) (Lu and Li 2020). Although *C. yunnanensis* might be confused with the Asian taxon *C. mirus*, the latter is distinct by its slender cylindrical, light brown basidiomes and broader basidiospores (Methven 1990). The presence of *C. mirus* in China needs to be ascertained. In the phylogenetic analyses, *C. yunnanensis* has a joint relationship with *C. elongatus*, *C. pistillaris* and the sequence of “*C. occidentalis*” from Tunisia, but the sister relationship cannot be resolved (Fig. 1).

### Taxonomic key to species of *Clavariadelphus* in China

**Table:** 

1	Basidiospores narrowly ellipsoid, *Q*_m_ > 2	**2**
–	Basidiospores broadly ellipsoid to ellipsoid, *Q*_m_ < 2	**3**
2	Basidiospores 11.0–14.0 × 4.0–5.5 μm, *Q*_m_ 2.7	***C. ligula***
–	Basidiospores 21–24 × 4–6 μm, *Q*_m_ 4.2	***C. sachalinensis***
3	Basidiomes orange; apex sterile, truncate	**4**
–	Basidiomes without orange tinge; apex fertile, not truncate	**5**
4	Basidiomata apex 3–7.5 cm diam	***C. amplus***
–	Basidiomata apex < 2 cm diam	***C. gansuensis***
5	Basidiomes usually 20–30 cm high	**6**
–	Basidiomes usually < 20 cm high	**7**
6	Basidiomes grey-purple; basidiospores narrowly ellipsoid, 9.0–11.0 × 5.7–7.4 μm, *Q*_m_ 1.71	***C. elongatus***
–	Basidiomes cinnamon; basidiospores broadly ellipsoid, 9.0–11.0 × 4.6–6.4 μm, *Q*_m_ 1.56	***C. yunnanensis***
7	Basidiomes greyish-red to pastel-red	***C. himalayensis***
–	Basidiomes grey or yellow, without red colouration	**8**
8	Basidiomes grey; basidiospores ellipsoid 10–11 × 5–6.5 μm, *Q*_m_ 1.89	***C. griseoclavus***
–	Basidiomes yellow colouration	**9**
9	Basidiomes yellow; basidiospores broadly ellipsoid 7.8–9.6 × 5.5–7.4 μm, *Q*_m_ 1.38	***C. alpinus***
–	Basidiomes pale yellow-brown; basidiospores narrowly ellipsoid 9.2–12.0 × 4.6–6 μm, *Q*_m_ 1.97	***C. khinganensis***

## Discussion

### The taxonomic importance of comprehensive data in *Clavariadelphus*

Many studies have verified that molecular methods are effective in resolving relationships in complicated groups of fungi (Zeng et al. 2013; Tang et al. 2017; Huang et al. 2018; Yang et al. 2018). In a pre-study analysis, we evaluated four DNA gene markers: ITS, large subunit of nuclear ribosomal RNA (nrLSU), translation elongation factor 1α gene (*tef1-α*) and DNA-directed RNA polymerase II second subunit (*rpb2*). Compared to the others, ITS offered the highest probability of successful identification in *Clavariadelphus*. By contrast, other markers displayed a lower success rate of PCR amplification or inferior species resolution in some close or sibling taxa. ITS sequences acquired from this study are listed in Appendix 1.

Macro-morphological, micro-morphological and SEM characteristics are very important in the taxonomy of *Clavariadelphus*. Although *Clavariadelphus* can be readily distinguished from other clavarioid genera, the delimitation of infrageneric taxa is difficult in many cases, especially without critical observation and examination (Methven 1990). Basidiomata colour is a diagnostic characteristic, although it must be used in conjunction with other morphological features. In China, basidiomata colour ranges from yellow to orange, grey-purple, pastel-red or brown. Despite the inherent variability in shape, size and other characteristics of the basidiomes, these features are important diagnostic characteristics in *Clavariadelphus*, as well as the size and shape of basidiospores. The basidiospores of Chinese taxa of this genus are summarised in Fig. 3 and 4. Additionally, some SEM characteristics, especially hyphae of the basal mycelium, are of taxonomic value. The variations of basal hyphae of Chinese taxa range from smooth, having nipple-shaped protuberances to crystals or both at the same time (Fig. 5).

Chemical reactions also are helpful in distinguishing *Clavariadelphus* species. Doty (1948) distinguished this genus using FeSO_4_ reactions. Corner reported that chemical reactions, especially KOH, are useful for delimiting *Clavariadelphus* taxa (Corner 1950). In this study, chemical colour reactions were conducted using seven chemical reagents (Table 1). Amongst these, four reagents were found to be discriminatory with some species, specifically KOH, FeCl_3_, NH_4_OH and phenol. Three additional reagents, namely ethanol, Melzer’s reagent and FeSO_4_, were found to lack discriminatory value.

Metadata supply taxonomic information, such as habit, distribution and host plants. The growth habit of Chinese taxa includes solitary, scattered and gregarious. Growth habit is of taxonomic value only when used in conjunction with other features (Methven 1990). The Chinese specimens were collected in mixed or coniferous forests in association with *Abies*, *Berberis*, *Quercus*, *Pinus*, *Picea*, *Rhododendron*, *Rosa*, *Salix*, *Thuja* and *Tsuga*. The distribution of a species usually correlates with that of its host plant. Although the Chinese taxa exhibit no apparent preference of host plants, the so-called cosmopolitan species within *Clavariadelphus* seem to be rare in this study.

### *Clavariadelphus* species diversity in China

Many new fungal taxa have been discovered in the last ten years in China (Zhang et al. 2005; Zeng et al. 2013; Tang et al. 2014; Huang et al. 2018; Yang et al. 2018). However, there are still a large number of undescribed fungal taxa in this country. This study indicates that there are at least ten known taxa of *Clavariadelphus* in China, including four previously described (*C. griseoclavus*, *C. ligula*, *C. sachalinensis* and *C. yunnanensis*), two not previously reported in China (*C. elongatus* and *C. himalayensis*) and four novel species (*C. alpinus* sp. nov., *C. amplus* sp. nov., *C. gansuensis* sp. nov. and *C. khinganensis* sp. nov.). Several taxa, previously reported from China, need to be confirmed, including *C. mirus*, *C. pistillaris* and *C. truncatus*. In China, there are still some species that need to be discovered, such as GenBank accession JQ991679. To date, with the four novel taxa described in this study, there are twenty-eight species of *Clavariadelphus* worldwide. Although the taxonomy of *Clavariadelphus* has received much attention in the past, this group needs to be further examined with molecular methods. More reliable sequence data, especially those species from North America and Europe, are needed to understand phylogenetic relationships better.

## Supplementary Material

XML Treatment for Clavariadelphus alpinus

XML Treatment for Clavariadelphus amplus

XML Treatment for Clavariadelphus elongatus

XML Treatment for Clavariadelphus gansuensis

XML Treatment for Clavariadelphus himalayensis

XML Treatment for Clavariadelphus khinganensis

XML Treatment for Clavariadelphus ligula

XML Treatment for Clavariadelphus sachalinensis

XML Treatment for Clavariadelphus yunnanensis

## Appendix 1

**Table:** Sequences used or produced in our phylogenetic analyses of Clavariadelphus in China.

Taxon	Voucher	Locality	GenBank Accession No. (ITS)
***Clavariadelphus alpinus***	**HKAS 57396**	**China, Yunnan**	**MK705888***
*C. americanus*	MycoMap # 1288	USA, Indiana	MK575228
*C. amplus*	HKAS 76577	China, Gansu	MK705851*
***C. amplus***	**HKAS 54876**	**China, Yunnan**	**MK705857***
*C. amplus*	HMAS 132008	China, Qinghai	MK705852*
*C. amplus*	HMAS 97090	China, Qinghai	MK705853*
*C. amplus*	HKAS 49229	China, Sichuan	MK705854*
*C. amplus*	HKAS 49278	China, Sichuan	MK705855*
*C. amplus*	HKAS 53797	China, Sichuan	MK705856*
*C. amplus*	HMAS 250466	China, Yunnan	MK705858*
*C. amplus*	HMAS 97248	China, Tibet	MK705859*
*C. amplus*	HMAS 59867	China, Tibet	MK705860*
*C. amplus*	HKAS 46160	China, Tibet	MK705861*
*C. amplus*	HKAS 46120	China, Tibet	MK705862*
*C. elongatus*	HKAS 76589	China, Gansu	MK705842*
*C. elongatus*	HKAS 50742	China, Sichuan	MK705843*
*C. elongatus*	HKAS 50801	China, Sichuan	MK705844*
*C. elongatus*	HMAS 260746	China, Yunnan	MK705845*
*C. elongatus*	HKAS 52425	China, Yunnan	MK705846*
*C. elongatus*	LAH 31397	Pakistan, Khyber Pakhtunkhwa	MG768847*
*C. elongatus*	SWAT 000559	Pakistan, Khyber Pakhtunkhwa	MG768848*
***C. gansuensis***	**HKAS 76487**	**China, Gansu**	**MK705847***
*C. griseoclavus*	BJTC FM964	China, Shanxi	MT302370
*C. griseoclavus*	BJTC FM965	China, Shanxi	MT302371
*C. himalayensis*	HKAS 50684	China, Yunnan	MK705863*
*C. himalayensis*	HKAS 58811	China, Yunnan	MK705864*
***C. khinganensis***	**MHHNU 7789**	**China, Inner Mongolia**	MK705865*
*C. khinganensis*	MHKMU H.Y. Huang 368	China, Jilin	MT447468*
*C. ligula*	HMAS 51688	China, Heilongjiang	MK705848*
*C. ligula*	HMAS 35954	China, Heilongjiang	MK705849*
*C. ligula*	HMAS 41146	Czech, –	MK705850*
*C. mucronatus*	OSC 1064138	USA, Oregon	EU526000
*C. occidentalis*	OSC 104664	USA, the Pacific Northwest	EU669308
*C. occidentalis*	OSC 112861	USA, the Pacific Northwest	EU669202
*C. occidentalis*	OSC 114250	USA, the Pacific Northwest	EU834202
*C. occidentalis*	OSC 114281	USA, the Pacific Northwest	EU846242
*C. occidentalis*	H21536	Tunisia, Aïn Draham	KU973835
*C. pistillaris*	NAMA 2017-123	USA, Wisconsin	MH979250
*C. pistillaris*	AMB 18611	Italy, Aquila	MT452507
*C. pakistanicus*	MH 129901	Pakistan, Khyber Pakhtunkhwa	HQ379937
*C. pakistanicus*	SR1742	India, –	MT012805
*C. sachalinensis*	MHHNU 7816	China, Inner Mongolia	MK705866*
*C. sachalinensis*	p061i	USA, the Pacific Northwest	EU624408
*C. sachalinensis*	p059i	USA, the Pacific Northwest	EU624410
*C. sachalinensis*	p058i	USA, the Pacific Northwest	EU624411
*C. sachalinensis*	OSC 96213	USA, the Pacific Northwest	EU834196
*Clavariadelphus* sp.	src121	USA, California	DQ974709
*Clavariadelphus* sp.	OSC 105674	USA, the Pacific Northwest	EU669206
*Clavariadelphus* sp.	HC-PNNT-268	Mexico, Mexico State	KT874982
*Clavariadelphus* sp.	ECM54	China, Zhejiang	JQ991679
*Clavariadelphus* sp.	MushroomObserver.org/254047	Mexico, Queteraro	MH304404
*Clavariadelphus* sp.	Montri-108	Switzerland, Montricher	MK028378
*C. subfastigiatus*	OSC 119587	USA, the Pacific Northwest	EU669207
*C. subfastigiatus*	MICH 73554	USA, Clackamas County	JX275756
*C. truncatus*	MA-Fungi 48062	Spain, –	AJ292288
*C. truncatus*	OUC99108	Canada, British Columbia	DQ097871
*C. truncatus*	SIM278	Canada, British Columbia	HQ650728
*C. truncatus*	AMB 18612	Italy, Belluno	MT452508
*C. unicolor*	Mushroom Observer #112193	USA, Indiana	MN906166
*C. yunnanensis*	HKAS 49398	China, Sichuan	MK705867*
*C. yunnanensis*	HKAS 31136	China, Sichuan	MK705868*
*C. yunnanensis*	HKAS 54849	China, Yunnan	MK705869*
*C. yunnanensis*	HKAS 63558	China, Yunnan	MK705870*
*C. yunnanensis*	HKAS 57731	China, Yunnan	MK705871*
*C. yunnanensis*	HKAS 58789	China, Yunnan	MK705872*
*C. yunnanensis*	HKAS 55244	China, Yunnan	MK705873*
*C. yunnanensis*	HMAS 250510	China, Yunnan	MK705874*
*C. yunnanensis*	HMAS 250471	China, Yunnan	MK705875*
*C. yunnanensis*	HKAS 62644	China, Yunnan	MK705876*
*C. yunnanensis*	HKAS 61417	China, Yunnan	MK705877*
*C. yunnanensis*	HKAS 43816	China, Yunnan	MK705878*
*C. yunnanensis*	HKAS 30752	China, Yunnan	MK705879*
*C. yunnanensis*	HKAS 30083	China, Yunnan	MK705880*
*C. yunnanensis*	HKAS 20068	China, Yunnan	MK705881*
*C. yunnanensis*	HKAS 20067	China, Yunnan	MK705882*
*C. yunnanensis*	HKAS 17788	China, Yunnan	MK705883*
*C. yunnanensis*	HKAS 15063	China, Yunnan	MK705884*
*C. yunnanensis*	HKAS 57659	China, Yunnan	MK705885*
*C. yunnanensis*	HKAS 77226	China, Yunnan	MK705886*
*C. yunnanensis*	HKAS 77288	China, Yunnan	MK705887*
*Lentaria byssiseda*	TENN 61159	USA, Tennessee	FJ596788
*Kavinia himantia*	CFMR:DLL2011-079	USA, Wisconsin	KJ140598
*K. alboviridis*	CFMR:DLL2011-131	USA, Wisconsin	KJ140634

* indicates sequences generated in this study, fontbold indicates type material for the new species.
